# Paramagnetic Ionic Liquid/Metal Organic Framework Composites for CO_2_/CH_4_ and CO_2_/N_2_ Separations

**DOI:** 10.3389/fchem.2020.590191

**Published:** 2020-11-16

**Authors:** Tiago J. Ferreira, Ana T. Vera, Beatriz A. de Moura, Laura M. Esteves, Mohammad Tariq, José M. S. S. Esperança, Isabel A. A. C. Esteves

**Affiliations:** Laboratório Associado para a Química Verde/Rede de Química e Tecnologia (LAQV/REQUIMTE), Departamento de Química, Faculdade de Ciências e Tecnologia, Universidade NOVA de Lisboa (FCT NOVA), Costa da Caparica, Portugal

**Keywords:** IL@MOF porous composites, gas adsorption, CO_2_ separation, ionic liquid (IL), metal–organic framework (MOF)

## Abstract

Global warming is arguably the biggest scientific challenge of the twenty-first century and its environmental consequences are already noticeable. To mitigate the emissions of greenhouse gases, particularly of CO_2_, there is an urgent need to design materials with improved adsorbent properties. Five different magnetic ionic liquids were impregnated into the metal–organic framework ZIF-8. The composites were produced by a direct-contact method, and their performance as sorbents for gas separation applications was studied. The impact of the ionic liquid anion on the sorption capacity and ideal CO_2_/CH_4_ and CO_2_/N_2_ selectivities were studied, focusing on understanding the influence of metal atom and ligand on the adsorbent properties. Reproducible methodology, along with rigorous characterization, were established to assess the impact of the ionic liquid on the performance of the composite materials. Results show that the ionic liquid was well-impregnated, and the ZIF-8 structure was maintained after ionic liquid impregnation. The produced composites were of microporous nature and were thermally stable. CO_2_, CH_4_, and N_2_ adsorption–desorption isotherms were obtained at 303 K and between 0 and 16 bar. The adsorption-desorption data of the composites were compared with that obtained for original ZIF-8. The general trend in composites is that the increased gas uptake per available pore volume compensates the pore volume loss. Adsorption data per unit mass showed that composites have reversible sorption, but inferior gas uptake at all pressure ranges. This is due to the observed total pore volume loss by the ionic liquid pore occupation/blockage. In most cases, composites showed superior selectivity performance at all pressure range. In particular, the composite [C_4_MIM]_2_[MnCl_4_]@ZIF-8 shows a different low-pressure selectivity trend from the original MOF, with a 33% increase in the CO_2_/N_2_ selectivity at 1 bar and 19% increase in the CO_2_/CH_4_ selectivity at 10 bar. This material shows potential for use in a post-combustion CO_2_ capture application that can contribute to greenhouse gas mitigation.

## Introduction

Around 90% of the anthropogenic atmospheric carbon dioxide (CO_2_) emissions, the most relevant greenhouse gas (GHG), are due to fossil fuel combustion processes (Jackson et al., 2017). The continuous misuse of highly pollutant energy sources is causing the accumulation of GHGs in the atmosphere, consequently contributing to global warming (GW) and environmental changes (Zheng et al., 2019). Between 2015 and 2040, atmospheric CO_2_ emissions are projected to grow at a yearly average rate of 0.6%. To mitigate the global mean temperature rise, the Paris Climate Agreement was set to limit the increase in temperature to a maximum of 2 °C compared to the pre-industrial level (Zhang et al., 2019). Therefore global efforts need to be set in practice, and this will involve the effective use of renewable fossil fuels substitutes, such as biomethane (bio CH_4_) upgraded from biogas (Lapa et al., 2017; Surra et al., 2019), as well as the stabilization of atmospheric CO_2_ concentrations at 450 ppm, which can be achieved through the carbon capture and storage (CCS) of 120–160 GtCO_2_ up to 2050 (Dowell et al., 2017).

The most established technology in the chemical industry for post-combustion CO_2_ capture from flue gas—a mixture mainly composed of nitrogen (N_2_) and CO_2_–is amine scrubbing. However, major drawbacks associated with the use of alkanolamines absorbents include their volatility, high regeneration input and equipment corrosion due to the presence of carbamates, formed upon the interaction of the solvent with CO_2_ (Theo et al., 2016). As a result, new materials are being considered for CCS technologies, such as gas absorption, gas adsorption or membrane permeation (Smit, 2016).

Ionic Liquids (ILs) are salts composed of asymmetric organic cations and organic or inorganic anions, with a conventional melting point below 373 K. These materials present interesting physical and chemical properties like negligible volatility, thermal stability, electrical conductivity and high CO_2_ solubility, having been intensively studied as alternative absorbents for performance improvement of gas absorption operations (Earle et al., 2006; Zeng et al., 2017). These materials are also referred as “designer solvents” given the almost unlimited number of potential structure combinations generated by the choice of anions and cations that allow tailoring their chemical interactions and physicochemical properties. Nowadays, besides their role as alternative absorbents in gas capture/separation, ILs are promising candidates to be used as electrolytes in batteries (Sun et al., 2019), fuel/lubricant additives (Zhou and Qu, 2017), solvents in biocatalysis (Egorova and Ananikov, 2018) and fillers in membrane processes (Sasikumar et al., 2018), among others.

In the last two decades, intensive studies have been conducted regarding Metal-Organic Frameworks (MOFs) as alternative adsorbents for gas adsorption (Trickett et al., 2017; Kochetygov et al., 2018; Hu et al., 2019). Adsorption-based processes are environmentally friendly, low-energy demanding and cost-effective when compared to more mature and deep-rooted CO_2_ capture/separation processes, such as amine scrubbing. MOFs are solid crystalline structured materials, due to the strong bonds between organic ligands and metal atoms/metallic clusters. The crystalline arrangement on these materials gives them a porous system with large specific surface areas that can surpass 6,000 m^2^/g, apart from high porosities along with thermal and chemical stabilities. Moreover, one can tune both the composition and topology of MOFs (Gangu et al., 2016; Hou et al., 2019). Literature shows that this class of materials has high CO_2_/CH_4_ and CO_2_/N_2_ selectivity performance, which makes MOFs promising materials for CO_2_ capture/separation processes (Li et al., 2014; Lin et al., 2016). Their large specific surface areas, designable pore sizes and surface functionality, obtained through different synthesis methods that influence their physical and chemical properties (Li et al., 2018b), allow these materials to be used in other fields apart from gas separation/storage (Camacho et al., 2015; Li et al., 2018a; Nabais et al., 2018; Ribeiro et al., 2019), such as catalysis (Liu et al., 2019), drug delivery (Cai et al., 2019), sensing applications (Fang et al., 2018), or ion exchange (Desai et al., 2018).

A new strategy to enhance the MOF material properties (namely, adsorption capacity and selectivity performance) involves the incorporation of ILs into their frameworks, forming new composite materials commonly known as IL@MOFs (Cota and Fernandez Martinez, 2017; Ferreira et al., 2019). Studies indicate that functionalizing MOFs with ILs creates different and stronger sorption sites for gas molecules which can provide enhanced gas affinity, vital in the development of new materials for adsorption-based processes (Kinik et al., 2016; Sezginel et al., 2016; Koyuturk et al., 2017; Mohamedali et al., 2018; Zeeshan et al., 2018). Given the multitude of existing MOFs and ILs, an almost infinite number of these sorbent composites can be produced for potential use as novel adsorbents with enhanced performance in gas separation applications.

In this work, 5 different paramagnetic ILs were impregnated in MOF ZIF-8 with the same molar loading. The CO_2_, CH_4_, and N_2_ adsorption capacities were determined for the magIL@ZIF-8 composites and compared with pristine ZIF-8. This crystalline MOF is composed of zinc ions coordinated by four 2-methylimidazolate rings. It has a high specific surface area and a porous network with pore cavities of 11.6 Å, along with pore windows/apertures of 3.4 Å (Zhang et al., 2018). ZIF-8 possesses particular high thermal stability and chemical resistance to both water and alkaline solutions/organic solvents (Du et al., 2016). The choice of these magnetic ionic liquids is based on the similarity with MOF ZIF-8 structure. They possess a metallic center in the anion and an imidazolium-based cation. The selection criteria of the magnetic ILs anion used to impregnate ZIF-8 were two-fold: (a) tetrachloro-based metal complex ILs to understand the effect of the metal coordination center of the anion; and (b) cobalt complex-based IL anions to determine the effect of different ligands. To the best of the authors knowledge, this is the first work regarding magnetic ILs impregnated into MOFs for gas adsorption applications.

These newly produced magIL@ZIF-8 materials were rigorously characterized to confirm the impregnation of the ILs and assess their textural properties, thermal and structural stabilities, as well as their surface morphologies. Several techniques were employed systematically in this work to fully characterize the composites prepared: static (isothermal) and dynamic (non-isothermal) thermogravimetric analysis (TGA); helium (He) pycnometry and N_2_ adsorption-desorption equilibrium at 77 K; Fourier transform infrared spectroscopy (FT-IR); powder X-ray diffraction (PXRD); scanning electron microscopy (SEM) and transmission electron microscopy (TEM). Moreover, single-component CO_2_, CH_4_, and N_2_ adsorption-desorption isotherms were obtained for the pristine ZIF-8 and the produced magIL@ZIF-8 materials at 303 K. For this purpose, it was employed a highly accurate standard static gravimetric method between 0 and 16 bar (Esteves et al., 2008). Ideal CO_2_/CH_4_ and CO_2_/N_2_ selectivities of ZIF-8 and magIL@ZIF-8 materials were determined and compared based on an accurate description of the obtained adsorption-desorption data.

## Materials and Methods

### Materials

**Figure 1:**
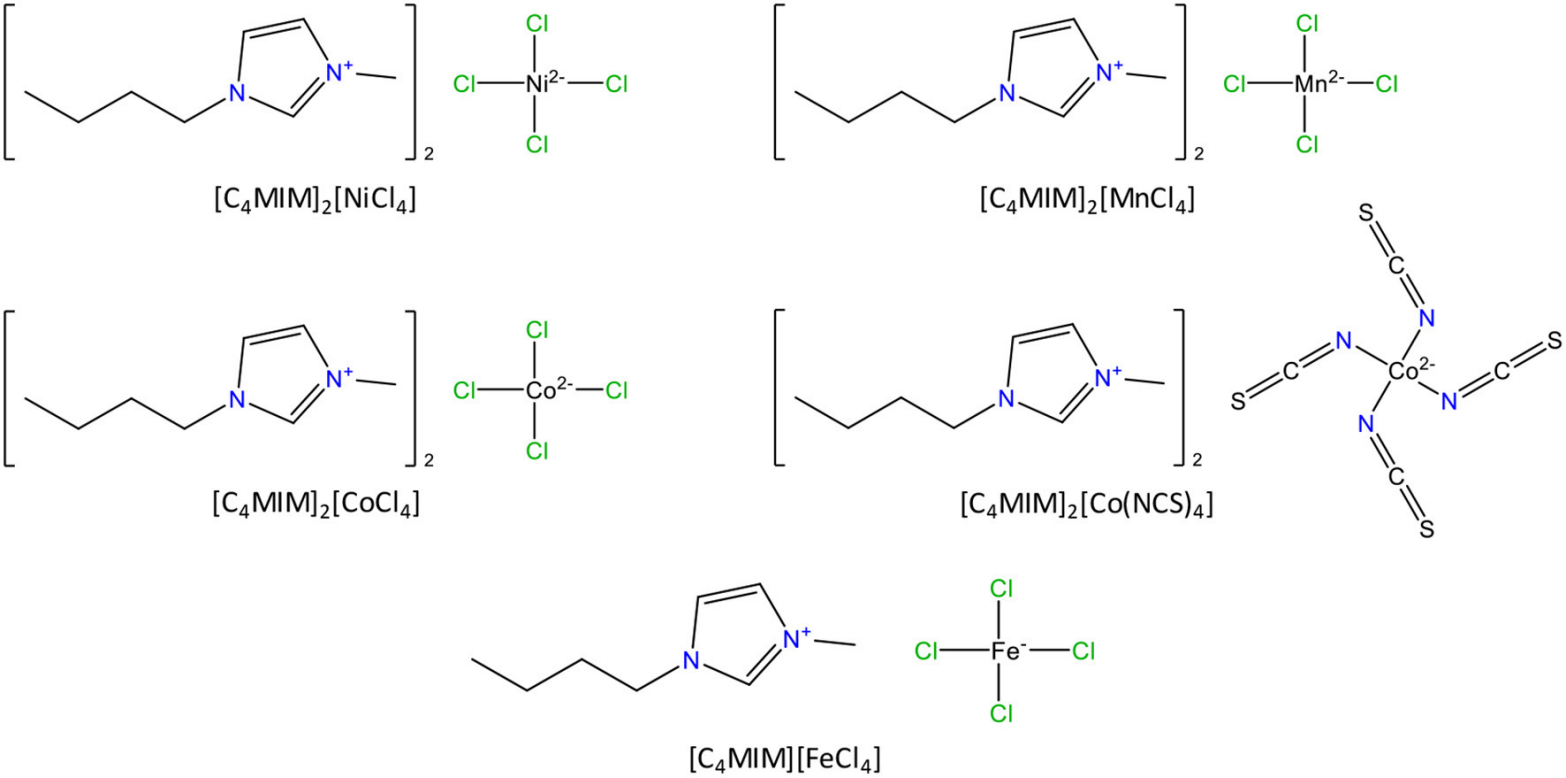
Chemical structures of the paramagnetic ionic liquids used in this work.

MOF ZIF-8 (Basolite® Z1200) was acquired from Sigma-Aldrich, as produced by BASF SE. [C_4_MIM]_2_[Co(NCS)_4_] (bis(1-butyl-3-methylimidazolium) tetraisothiocyanatocobaltate, >99%) was purchased from IoLiTec. ILs [C_4_MIM]_2_[NiCl_4_] (bis(1-butyl-3-methylimidazolium) tetrachloronickelate, >99%), [C_4_MIM]_2_[MnCl_4_] (bis(1-butyl-3-methylimidazolium) tetrachloromanganate, >99%), [C_4_MIM]_2_[CoCl_4_] (bis(1-butyl-3-methylimidazolium) tetrachlorocobaltate, >99%) and [C_4_MIM][FeCl_4_] (1-butyl-3-methylimidazolium tetrachloroferrate, >99%) were synthesized and purified in-house. Their synthesis protocol can be found elsewhere (Zhong et al., 2007; Meredith et al., 2009; Cruz et al., 2013). Their chemical structures are represented in Figure 1. All ILs were evacuated at 0.1 Pa and 323 K for at least 24 h, prior to any material preparation. Acetone (99.8%, Carlo Erba) and ethanol (99.9%, Scharlau) were used as solvents for ILs dissolution and to promote their impregnation into the MOF structure. Ethanol was used to dissolve [C_4_MIM]_2_[NiCl_4_], [C_4_MIM]_2_[MnCl_4_] and [C_4_MIM]_2_[CoCl_4_] while acetone was used for the remaining two ILs. Ethanol was also used in sample preparation for STEM imaging. Potassium bromide (KBr, Panreac) was employed in the preparation of FT-IR pellets of solid samples. The gases N_2_ (> 99.998%), CH_4_ (99.95%), CO_2_ (99.998%), and He (≥ 99.999%), used in the adsorption-desorption equilibria measurements, were purchased from Praxair.

### Samples Preparation

The preparation of magIL@ZIF-8 composites followed the same experimental protocol and IL molar loading (9.3 mol%) reported in our previous work (Ferreira et al., 2019). Summarily, the IL was weighed in a vial and dissolved in acetone/ethanol. This mixture was then stirred at room temperature for 15 min. Afterwards, the IL solution was added to 1 g of degassed ZIF-8 (373 K for 3–4 h), previously weighed in another vial. The mixture was then capped and stirred overnight at room temperature. After that, the cap was removed, and the mixture continued stirring for 4–5 h at room temperature and later at 338 or 353 K (when the solvent was acetone or ethanol, respectively) to promote solvent evaporation. Finally, the sample was degassed at 373 K (3–4 h to remove trace solvent molecules) and stored in a desiccator.

### Samples Characterization

#### Thermogravimetric Analyses (TGA)

Thermogravimetric analyses of pristine ZIF-8, neat ILs and magIL@ZIF-8 composites were performed using a thermogravimetric analyzer Labsys EVO (Setaram). The sample was held in an alumina sample pan, under a 50 mL/min flow of argon (Ar). The analyses were performed from room temperature up to 1,073 K, with a heating rate of 2 K/min. An isothermal treatment of the samples was performed *in situ*, at 373 K for 1 h, to remove moisture or other pre-adsorbed impurities. This procedure assures a completely dry sample prior to the dynamic analysis, and therefore the observed mass loss is exclusively due to structural decomposition with temperature.

#### N_2_ Adsorption-Desorption Equilibrium at 77 K

A static volumetric apparatus ASAP 2010 (Accelerated Surface Area and Porosimetry System, Micromeritics) was used to perform N_2_ adsorption-desorption equilibrium at 77 K. Before the measurement, ZIF-8 and magIL@ZIF-8 composites were degassed under vacuum for 3–4 h at 373 K. Different textural properties of the materials were obtained, along with the pore size distributions (assuming a slit-shaped pores model).

#### Powder X-ray Diffraction (PXRD)

The powder X-ray diffractograms of ZIF-8 and magIL@ZIF-8 materials were obtained using an X-ray diffractometer MiniFlex II (Rigaku). The X-ray generator has 30 kV of voltage and 15 mA of current. A Cu radiation source was used, and analyses were carried out between 2θ values of 2° and 50°, with a scanning speed of 0.5°/min and a step width of 0.02° for all samples.

#### Fourier Transform Infrared (FT-IR) Spectroscopy

FT-IR spectra of all the materials used in this work were performed in a FT-IR Spectrometer Spectrum Two model (PerkinElmer). Neat ILs spectra were obtained through an Attenuated Total Reflectance (ATR) modulus, while the spectra of solid samples (ZIF-8 and magIL@ZIF-8 composites) required a Transmittance modulus. For the latter materials, it was required the preparation of KBr pellets that contained some milligrams of sample. Measurements were performed at room temperature conditions, between 4,000 and 450 cm^−1^, with a spectral resolution of 4 cm^−1^.

#### Scanning and Transmission Electron Microscopy (STEM)

SEM and TEM imaging of ZIF-8 and magIL@ZIF-8 materials were obtained with an UHR-STEM HD2700 type B model (Hitachi) with an acceleration voltage of 200 kV. Prior to the obtention of the microscopic images, a general sample preparation procedure was followed. Firstly, a few milligrams of material were added to an Eppendorf with ethanol. The content of the Eppendorf was mixed, and some time was given for the deposition of the suspended particles. Finally, a drop of this suspension was added to a carbon-coated copper grid (400 mesh), which was dried at room temperature. SEM and TEM imaging were obtained at different magnifications, ranging from 15k x to 150k x.

#### Gas Adsorption-Desorption Equilibria of CO_2_, CH_4_, and N_2_

**Figure 2:**
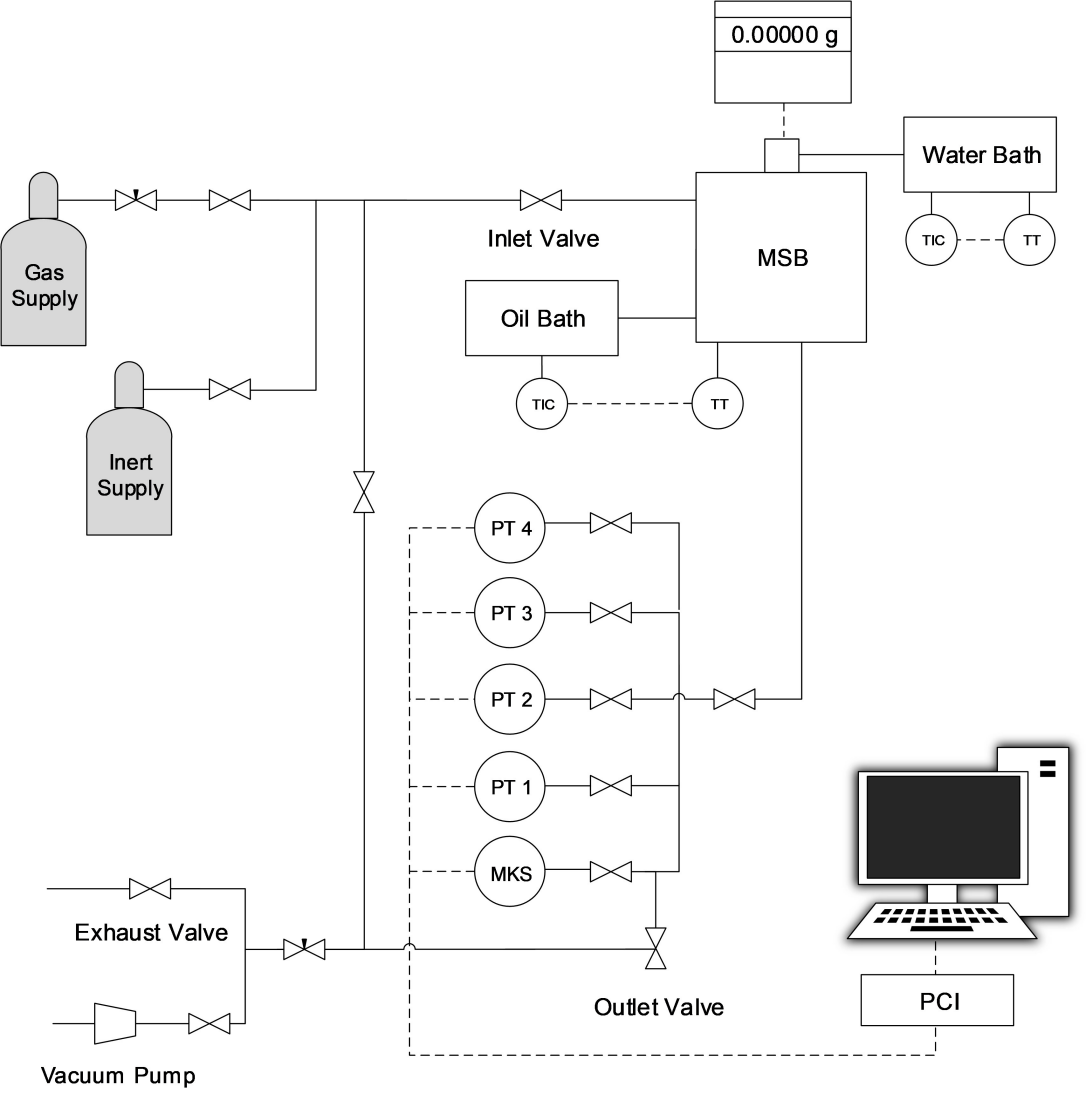
Schematic representation of the experimental setup for adsorption-desorption equilibrium measurements: MSB - magnetic suspension balance; PT - Omegadyne pressure transducer; MKS-MKS Baratron pressure transducer; PCI -PC interface for data acquisition.

Adsorption-desorption equilibria of CO_2_, CH_4_, and N_2_ in ZIF-8 and in all magIL@ZIF-8 composites were experimentally measured through a standard static gravimetric method, at 303 K and in a pressure range of 0–16 bar. The schematic of the experimental setup is depicted in Figure 2. The main core of the unit is a high accuracy ISOSORP 2000 magnetic-suspension balance (Rubotherm GmbH). The balance has a resolution of 10^−5^ g, uncertainty ≤ 0.002% and reproducibility ≤ 3 × 10^−5^g. Temperature inside the unit is controlled using an accurate F32-HL (Julabo GmbH) heating/refrigerating circulator (±0.1 K). Pressure measurements are made using a parallel set of pressure transducers that ensure accurate readings over all operational pressure ranges. In this work, a Baratron model 627D (MKS Instruments GmbH, accurate to 0.12% of the measured value) and Omegadyne Inc. models PX01C1-150A5T and PX01C1-500A5T (PT 2 and PT 3 of Figure 2, both accurate to 0.05% of full scale) were used for pressure measurements between 0-1 bar, 0-10 bar and 0-35 bar, respectively. The pressure recording and monitorization was done using an in-house developed software. Measurements were performed using 0.3–0.4 g of material and, prior to these, the apparatus was checked for gas leaks. After this check, the samples were degassed *in situ* at 373 K for 3–4 h, under vacuum and then conditioned overnight to 303 K, under vacuum. Replicate measurements were done to check data reproducibility. Further details regarding the gravimetric unit and methodology employed in the measurements can be found elsewhere (Esteves et al., 2008; Camacho et al., 2015).

For a given gas of interest, its adsorbed amount is usually reported in mass or moles of adsorbed gas per mass of adsorbent. Net adsorption quantity (Gumma and Talu, 2010), *q*_net_, can be used to report adsorption data. Considering a gravimetric experiment, the specific net amount adsorbed, in moles of adsorbed gas per mass of adsorbent, is obtained by

(1) $$\begin{array}{r} q_{\text{net}}=\frac{m-m_{\text{s}}-m_{\text{h}}+V_{\text{h}}\;\rho_{\text{g}}}{m_{\text{s}}\;M_{\text{w}}} \end{array}$$

where *m* is the weighed mass, *m*_s_ is the mass of (degassed) solid, *m*_h_ and *V*_h_ are the total mass and volume of all parts of the sample holder contributing to buoyancy effects, respectively; ρ_g_ is the density of the gas at the equilibrium pressure and temperature and *M*_w_ is the molecular weight of the adsorbed gas. The report of net adsorption data avoids drawbacks concerning the use of probe molecules for the determination of the reference state. However, most adsorption studies still report adsorption data in total adsorption quantity (*q*_t_); thus *q*_net_ can be converted to *q*_t_, in moles of adsorbed gas per mass of adsorbent, by

(2) $$\begin{array}{r} q_{\text{t}}=q_{\text{net}}\;+\;\left(V_{\text{p}}+V_{\text{s}}\right)\;\rho_{\text{g}} \end{array}$$

where *V*_p_ is the adsorbent total pore volume (determined by N_2_ adsorption-desorption equilibrium at 77 K) and *V*_s_ is the volume of adsorbent that is impenetrable to adsorbate molecules. The solid matrix density (ρ_s_ = 1/*V*_s_) was determined by He pycnometry. The above formula can still be applied when a porous solid is impregnated with IL, though both *V*_p_ and ρ_s_ must be corrected for the presence of the IL. The He pycnometry measurements were performed using the same gravimetric method at 333 K. For this technique, He is assumed as an inert probe molecule (does not adsorb) that penetrates all the accessible pore volume of the adsorbent.

## Results and Discussion

### Thermogravimetric Analyses (TGA)

For the evaluation of the thermal stability of the pristine ZIF-8, neat ILs and magIL@ZIF-8 composites, TGA data were obtained (see Supplementary Figure 1). The ZIF-8 thermogram shows a single stage of mass loss, consistent with the decomposition of 2-methylimidazole, the organic linker of ZIF-8 (James and Lin, 2016). The neat tetrachloro-based metal complex ILs shows two decomposition stages: the first one is related to the decomposition of the imidazolium cation(s), and the second one is consistent with the degradation of the anion (Meredith et al., 2009). A final constant weight stage can be found for these ILs, where element metal and metallic chloride salts are the remaining products (Meredith et al., 2009). The neat [C_4_MIM]_2_[Co(NCS)_4_] IL shows only one decomposition stage. This stage is therefore consistent with the decomposition of the imidazolium cations, followed by a very slow anion degradation. The magIL@ZIF-8 composites show the decomposition stage(s) of their neat IL counterpart, along with the organic linker decomposition stage of ZIF-8.

The starting decomposition temperature, *T*_start_, was considered for the temperature at which sample mass loss reaches 1 wt.%. The experimental results are shown in Table 1. Significant differences can be found between the *T*_start_ of some neat tetrachloro-based metal complex ILs and their respective magIL@ZIF-8 composites. However, both Co-based composites do not show a significant difference in its *T*_start_ relative to its neat IL counterpart. Therefore, both the metal center and ligand present in the anionic metal complex can affect the thermal stability of the magIL@ZIF-8 composites.

**Table 1:** Starting decomposition temperatures, IL weight loading, and remaining sample amount at 1,073 K of all the materials used and produced in this work, obtained by TGA.

**Sample**	***T*_**start**_ (K)**	**IL loading (wt.%)**	**Experimental remaining**	**Calculated remaining sample**
			**sample amount (wt.%)**	**amount (wt.%)^a^**
ZIF-8	690	0.0	63	—
Neat [C_4_MIM]_2_[NiCl_4_]	531	—	11	—
[C_4_MIM]_2_[NiCl_4_]@ZIF-8	460	17.7	52	54
Neat [C_4_MIM]_2_[MnCl_4_]	510	—	21	—
[C_4_MIM]_2_[MnCl_4_]@ZIF-8	456	17.6	50	56
Neat [C_4_MIM]_2_[CoCl_4_]	534	—	6	—
[C_4_MIM]_2_[CoCl_4_]@ZIF-8	538	17.7	52	53
Neat [C_4_MIM]_2_[Co(NCS)_4_]	529	—	19	—
[C_4_MIM]_2_[Co(NCS)_4_]@ZIF-8	533	20.4	53	54
Neat [C_4_MIM][FeCl_4_]	591	—	10	—
[C_4_MIM][FeCl_4_]@ZIF-8	471	13.2	52	56

a *Calculated from Equation (3), at 1,073 K*.

From the experimental thermograms of ZIF-8, neat ILs and mass loading percentages, it is possible to obtain calculated TGA profiles of the composites, as shown in Table 1.

(3) $$\begin{array}{r} {\text{Calc}.\text{wt}.\text{\% }}_{\text{at T}}=\text{wt}.{\text{\%}}_{\text{ ZIF}-8\text{ at T}}\;\times \;\left(1-\text{wt}.\text{\% loadin}{\text{g}}_{\text{ IL}}\right)\\ +\text{ wt}.\text{\%}{\;}_{\text{IL at T}}\;\times \text{ wt}.\text{\% loading}{\;}_{\text{IL}} \end{array}$$

These calculated profiles were compared with the experimental ones; it is observed in Supplementary Figure 1 that in general there is a higher thermal degradation rate than it was expected (as the blue thermograms are mostly shifted to the left of the green ones). This can be explained by the presence of the ILs (and respective degradation products) that accelerate the degradation of ZIF-8. This suggests the existence of direct and specific IL-MOF interactions.

It is also possible to compare experimental and calculated remaining composite amounts. Considering the TGA profiles of ZIF-8 and respective IL, as shown in Table 1, most composites show very close remaining amounts to what it was expected. When compared to other composites, a lower experimental remaining amount for [C_4_MIM]_2_[MnCl_4_]@ZIF-8 and [C_4_MIM][FeCl_4_]@ZIF-8 was obtained. This can also be explained by the interactions between the IL and ZIF-8, the former hastening the decomposition of the latter.

### Textural Properties of ZIF-8 and magIL@ZIF-8 Composites

The standard technique used to determine the textural properties of the materials is N_2_ adsorption-desorption equilibrium at 77 K. From this technique, as presented in Table 2, textural properties including BET, Langmuir specific surface areas (*A*_BET_ and *A*_Langmuir_, respectively), total pore volume (*V*_p_) and micropore volume of materials (*V*_micropore_, calculated from the Dubinin-Astakhov equation) were determined. Solid matrix density (ρ_s_) was estimated by He pycnometry. The total pore volume of the materials was defined for a relative pressure (*p*/*p*_0_) of 0.97, where *p* and *p*_0_ corresponds to the adsorbate equilibrium and saturation pressures, respectively. The ZIF-8 textural properties are in agreement with those reported in the literature (Park et al., 2006; Cao et al., 2013; Toyao et al., 2015). The obtained isotherms, shown in Supplementary Figures 2, 3, are all Type I, according to the IUPAC classification (Thommes et al., 2015) which indicates that ZIF-8 and the derived composites are microporous materials. It was not observed any hysteresis which means that the materials are completely reversible regarding N_2_ adsorption at 77 K.

**Table 2:** Textural properties of ZIF-8 and magIL@ZIF-8 composites.

**Sample**	***V*_**p**_**	***V*_**micropore**_**	***A*_**BET**_**	***A*_**Langmuir**_**	**ρ_**s**_**
	**(cm^**3**^/g)**	**(cm^**3**^/g)**	**(m^**2**^/g)**	**(m^**2**^/g)**	**(cm^**3**^/g)**
ZIF-8	0.672	0.655	1862	1926	1.49
[C_4_MIM]_2_[NiCl_4_]@ZIF-8	0.296 (0.360)	0.281 (0.341)	768 (933)	788 (958)	1.35
[C_4_MIM]_2_[MnCl_4_]@ZIF-8	0.210 (0.254)	0.205 (0.249)	551 (668)	580 (704)	1.37
[C_4_MIM]_2_[CoCl_4_]@ZIF-8	0.242 (0.294)	0.232 (0.282)	643 (782)	665 (808)	1.24
[C_4_MIM]_2_[Co(NCS)_4_]@ZIF-8	0.278 (0.349)	0.267 (0.335)	731 (919)	764 (960)	1.34
[C_4_MIM][FeCl_4_]@ZIF-8	0.237 (0.273)	0.230 (0.264)	639 (736)	657 (756)	1.33

*The values in brackets are in IL-free basis, expressed per gram of ZIF-8*.

As observed in Table 2, the total pore volume loss is between 56 and 69% after IL impregnation, with the same trend found for the BET and Langmuir specific surface areas—respective losses of 57–69 and 59–70%. This is attributed to the presence of the IL in the ZIF-8 framework, as these properties are related to the amount of ZIF-8 present in each composite. Per gram of MOF, a variable amount of IL is found in the composites. Therefore, to realize the IL impact in the composite, textural properties were calculated per gram of ZIF-8 (IL-free basis, see Table 2). Given the similar mass and molar loadings of the dicationic ILs, it was expected that the textural properties of the corresponding composites would be nearly identical. However, the ionic radius increases in the order Ni^2+^ < Co^2+^ < Fe^2+^ < Mn^2+^. Since the same molar amount of IL was impregnated, an anion with a smaller metallic center will block/occupy less ZIF-8 pore volume. This explains the trends in IL-free basis for the different textural properties (Table 2). It should be noted that the classical BET data treatment is performed for relative pressures between 0.05 and 0.30. For highly microporous materials, the low-pressure range should be extended for the calculations (Thommes et al., 2015). Some authors prefer characterizing these materials with Langmuir analysis; BET and Langmuir specific surface areas are very similar, with Langmuir values slightly superior.

Non-local density functional theory (NLDFT) calculations were performed with the N_2_ adsorption-desorption at 77 K data, from which the pore size distribution (PSD) of ZIF-8 and magIL@ZIF-8 materials were obtained (see Supplementary Figure 4). The composites present a lower total available pore volume than the pristine ZIF-8, and no pores larger than 20 Å were found, confirming the microporous nature of these materials.

### Powder X-ray Diffraction (PXRD)

PXRD patterns were obtained to evaluate the crystallinity of the magIL@ZIF-8 materials (see Supplementary Figure 5). The ZIF-8 PXRD data are in accordance with other patterns of this material found in the literature (Kinik et al., 2016). From the collected data, there is little to no change in the crystalline structure of the MOF after IL impregnation, as the peaks found in the diffractogram of ZIF-8 are also found in the composite materials. Besides this, every peak in each composite pattern has low intensity when compared with the ZIF-8 one, which can be explained by the IL presence in the ZIF-8 structure. This causes changes in the electronic density of the MOF, as has been observed in other IL@MOF materials (Mohamedali et al., 2018). From this technique, it is confirmed that the impregnation method used in this work did not compromise the crystalline structure of the MOF. Thus, every composite is also a crystalline material.

### Fourier Transform Infrared (FT-IR) Spectroscopy

**Figure 3:**
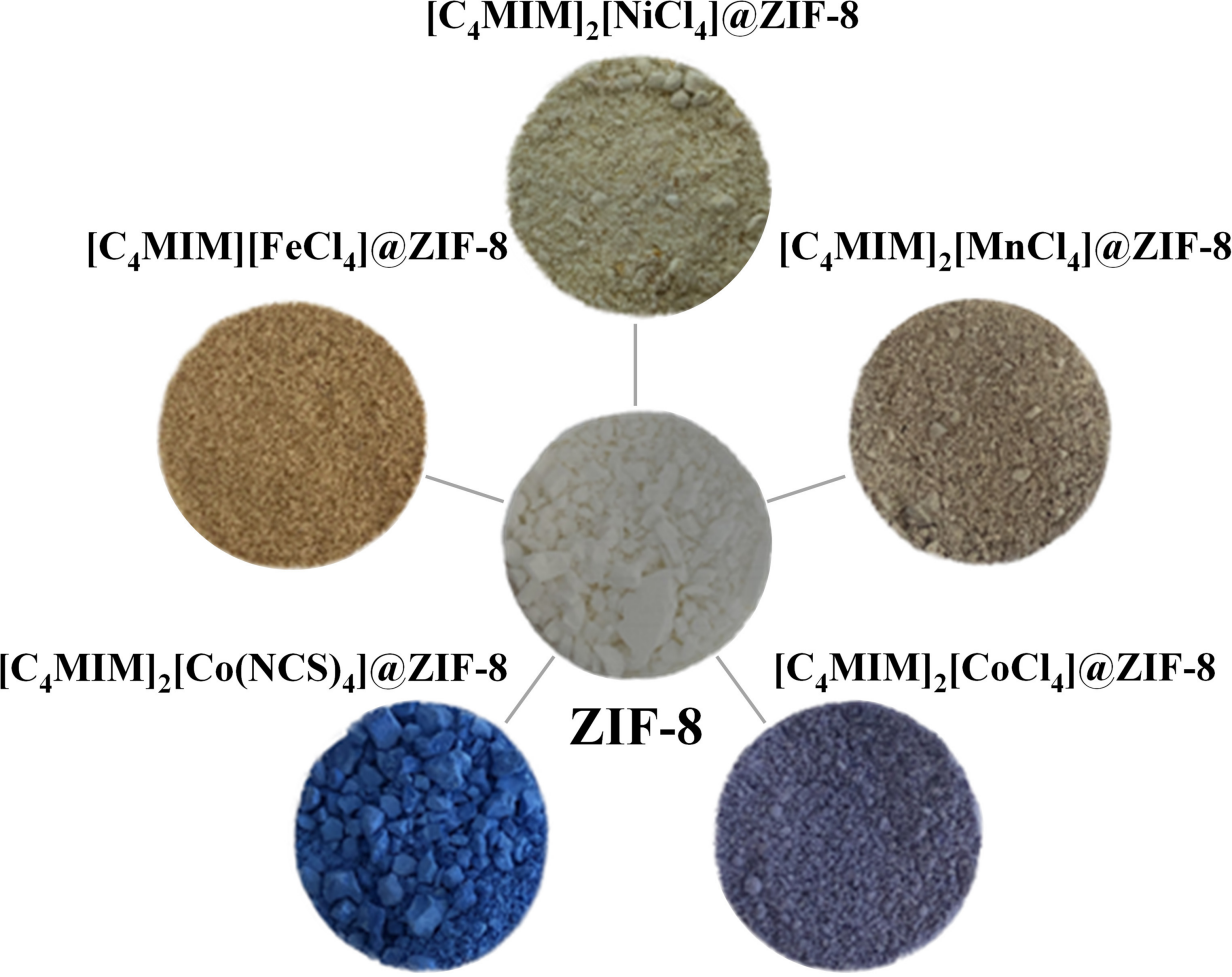
Samples of ZIF-8 and produced magIL@ZIF-8 powdered materials.

The IL incorporation into the structure of ZIF-8 was qualitatively checked by FT-IR. As composites, their FT-IR spectrum should include MOF-related and IL-related bands; the latter ones confirming the presence of IL in the structure of ZIF-8. FT-IR spectra of ZIF-8, neat ILs and magIL@ZIF-8 materials can be seen in Supplementary Figures 6–10. The ZIF-8 FT-IR spectrum is consistent with others found in the literature (Kinik et al., 2016; Koyuturk et al., 2017). The IL [C_4_MIM]_2_[Co(NCS)_4_] has bands at 2,050 cm^−1^ (CN stretching), 830 cm^−1^ (CS stretching), and 479 cm^−1^ (NCS bending) (Kabešová and Gažo, 1980). With these bands, it is possible to confirm that the anionic Co complex has four N-bonded monodentate ligands. Its respective composite has an IL-related band at 2,070 cm^−1^, a blue shift of 20 cm^−1^. A possible interpretation of this shift is that the IL anion interacts with the organic linkers of ZIF-8 (imidazolium rings), receiving electrons. This changes the electronic density in the composite, as indicated by PXRD data. This significant band shift confirms existing IL-MOF interactions, as previously disclosed by TGA. As for the tetrachloro-based metal complex composites, they do not present evident IL-related bands. This is expected, as M-Cl stretching bands (M stands for metal) have been reported in the literature below or near the used FT-IR spectrometer minimum range (450 cm^−1^) (Thompson et al., 1982; Chu et al., 2001; Sathyanarayana, 2005). However, given the negligible volatility of ILs and the fact that metal complexes usually present color, the IL impregnation can be visually perceived. All these magnetic ionic liquids are colored; after impregnation, as observed in Figure 3, the composites are also colored, compared with the white original ZIF-8 powder.

### Scanning and Transmission Electron Microscopy (STEM)

**Figure 4:**
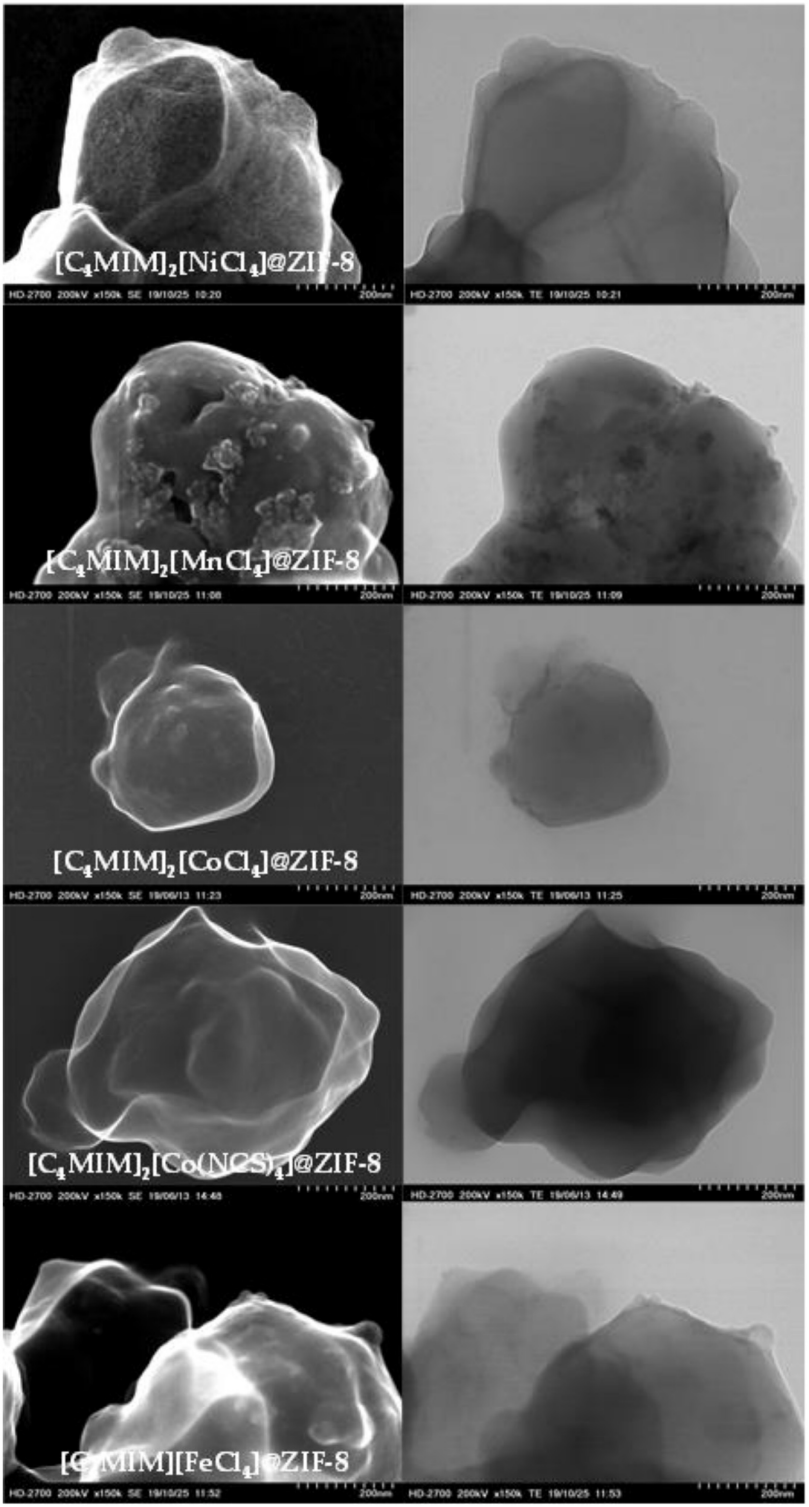
SEM (left) and TEM (right) imaging of magIL@ZIF-8 composites (150k x magnification and 200 nm scale).

Microscopic imaging of the pristine ZIF-8 and produced magIL@ZIF-8 composites were obtained from SEM and TEM, in order to analyze the surface morphology of these materials (Figure 4 and Supplementary Figures 11–15). The surface morphology of ZIF-8 is in agreement with the literature (Kinik et al., 2016) and its images obtained by the group can be found elsewhere (Ferreira et al., 2019). The micrographs presented in Figure 4 show that the MOF crystals tend to be more rounded upon IL impregnation, while maintaining crystalline structures. This is consistent with the obtained PXRD results. ILs seem to be at the outer surface of the ZIF-8 framework. In addition, the ILs seem to interact differently with the MOF, depending on the anionic coordination center. For instances, the [C_4_MIM]_2_[MnCl_4_]@ZIF-8 surface morphology is quite distinct from the other composites. It cannot be detected an effect of the ligand, as there is no discernible difference between the [C_4_MIM]_2_[CoCl_4_]@ZIF-8 and [C_4_MIM]_2_[Co(NCS)_4_]@ZIF-8 surface morphologies.

### Adsorption Performance of the magIL@ZIF-8 Composites

There are several factors in the synthesis route of magIL@MOF composites that impact on their gas adsorption performance. While the MOF/IL combination is crucial, other features such as the solvent or total pore volume loss due to IL impregnation can also affect the gas uptake in the composites. In our previous study, it was reported that acetone-washed ZIF-8 presents the same crystalline structure and gas uptake as pure ZIF-8 (Ferreira et al., 2019). Since some ILs did not dissolve in acetone, ethanol was also used herein as an IL impregnation promoting solvent in the method to prepare the composites. A sample of ZIF-8 was dissolved in ethanol and stirred overnight. This sample was named “ethanol-washed ZIF-8” and was characterized by TGA, PXRD, and FT-IR analyses. The obtained results were nearly identical to ZIF-8 (see Supplementary Figures 16–18). Furthermore, pure component CO_2_ and CH_4_ adsorption-desorption equilibrium isotherms were measured at 303 K and compared with pristine ZIF-8 through net adsorption quantity. The obtained results show that the use of ethanol as impregnation promoting solvent does not affect the uptake of both gases in ZIF-8 (see Supplementary Figure 19).

Single-component CO_2_, CH_4_, and N_2_ adsorption-desorption equilibria isotherms were measured at 303 K in the magIL@ZIF-8 composites and compared with the pristine ZIF-8 (see Supplementary Tables 1–6). The experimentally obtained isotherms are all Type I, according to IUPAC classification (Thommes et al., 2015). For all the materials, CO_2_ was more adsorbed than CH_4_ and N_2_, because of its significant quadrupole moment (Chiang et al., 2019). This leads to stronger attraction to the ZIF-8 framework and higher solubility in ILs.

The isotherms found in Supplementary Figure 20 show that, for all gases and pressure ranges, magIL@ZIF-8 composites adsorb less than the pristine ZIF-8. This is consistent with the total pore volume and specific surface area losses observed in the composites through the N_2_ adsorption-desorption measurements at 77 K (Table 2). At the highest pressure (16 bar), the loss of adsorption capacity when compared to the pure ZIF-8 was between 47–67% for N_2_, 39–65% for CH_4_ and 36–42% for CO_2_, per gram of composite.

Given that the available pore volume is the dominant factor in gas uptake at moderate to high pressure ranges, the obtained results were somewhat expected. In the low-pressure range (0–1 bar), all composites also show less adsorption capacity than the pristine ZIF-8. This means that the interactions, which are the dominant factor at this pressure range, between MOF, IL, and adsorbate are not strong enough to overcome the loss of adsorption sites. Specifically, IL-adsorbate interactions must have a physical character (that was expected given the ILs structure), which means that the new “sorption” sites that were created upon IL impregnation are relatively weak. Note that the term “sorption” includes both phenomena of gas adsorption and gas absorption. As previously mentioned, the selection criteria of ILs in this work was related to their structure. In tetrachloro-based metal complex ILs, the adsorption capacity decreases at all pressure ranges in the following order: [CoCl_4_]^2−^ > [NiCl_4_]^2−^ > [FeCl_4_]^−^ > [MnCl_4_]^2−^ for CO_2_ and CH_4_, and [CoCl_4_]^2−^ > [FeCl_4_]^−^ > [NiCl_4_]^2−^ > [MnCl_4_]^2−^ for N_2_ (see Supplementary Figure 20). The observed differences in gas uptake are not directly explained by a single property of the ILs. Instead, it is suggested that a combination of bond polarity and anion interaction with ZIF-8/adsorbate could explain the observed trends. Given that all these gases are apolar, bond polarity could help explain why the [C_4_MIM]_2_[MnCl_4_]@ZIF-8 composite adsorbs less, since it has the most polar anionic bonds of the four tetrachloro-based ILs. However, bond polarity does not explain the tendencies relative to [C_4_MIM]_2_[CoCl_4_]@ZIF-8 and [C_4_MIM]_2_[NiCl_4_]@ZIF-8, as they have similar anionic bond polarity. Specific interactions between the anion of these ILs and ZIF-8 (particularly its metal site), along with the different gas solubilities, can also explain these trends.

Regarding the ligand effect in cobalt complex-based ILs, it is clear that [Cl]^−^ is a better ligand for gas uptake than [NCS]^−^. The presence of sulfur (along with not being the donor atom) grants an acidic character to the anion, which is not favorable for neutral or slightly acidic gases sorption, such as the ones used in this work (Álvarez et al., 2017).

**Figure 5:**
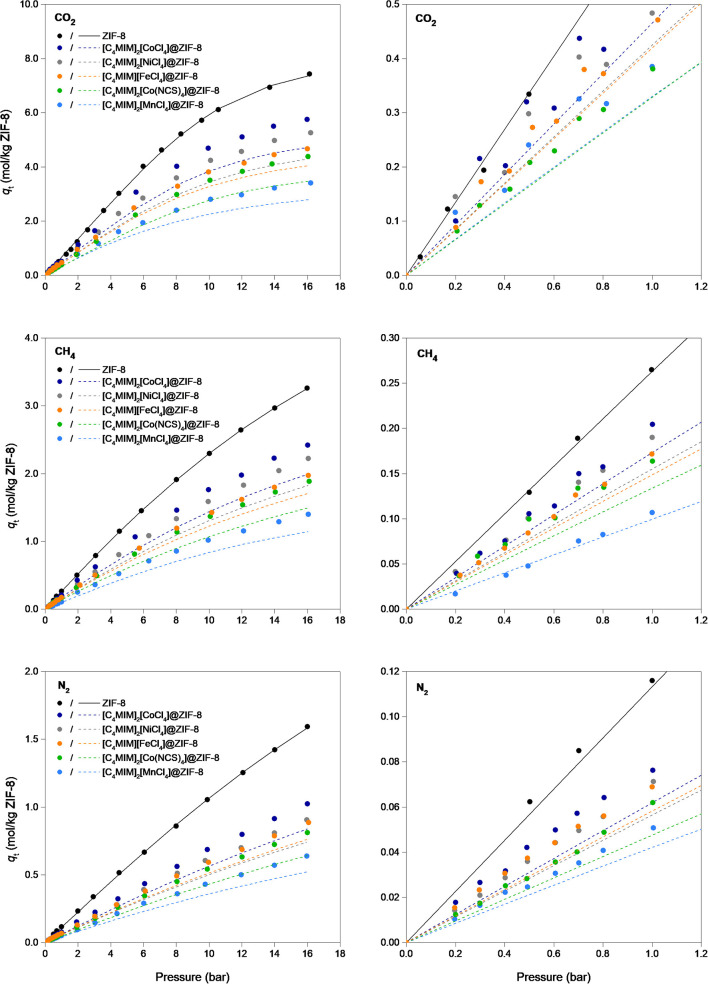
(Left) Single-component adsorption–desorption equilibrium isotherms of CO_2_, CH_4_, and N_2_ on an IL-free basis in pristine ZIF-8 and the magIL@ZIF-8 composites at 303 K. Symbols denote the adsorption and desorption data. Lines represent the Toth adsorption isotherm model fitting (Do, 1998) to the experimental non-IL-free data points; (Right) a low-pressure range inset of the data is shown.

The isotherms presented in Supplementary Figure 20 are expressed in moles of adsorbed gas per gram of composite. To properly compare the gas uptake of the composites with the pristine ZIF-8, an IL-free basis can be considered, normalizing the adsorbed amount per gram of ZIF-8 in the composite. The CO_2_, CH_4_, and N_2_ IL-free isotherms are found in Figure 5. Results show that the proper selection of the IL is crucial for the gas adsorption performance of these composite materials. As the same number of IL moles were impregnated, IL-free basis adsorption-desorption equilibria isotherms for a given gas would collapse into one. Since this does not happen, the structure of the IL (in particular, the anion) dictates the adsorption capacity in magIL@MOF materials, along with the IL gas solubility and incorporation mechanism into the MOF framework. Using this basis, it can be observed that the descending order of anions, for all gases, in terms of adsorption capacity at high pressure is [CoCl_4_]^2−^ > [NiCl_4_]^2−^ > [FeCl_4_]^−^ > [MnCl_4_]^2−^. At low pressure, this trend can change depending on the gas. This confirms that the choice of the coordination center plays a role for adsorption purposes, along with the choice of the ligand as [Cl]^−^ > [NCS]^−^. In addition, in an IL-free basis, the lower gas adsorption capacities of the magIL@ZIF-8 materials can also be explained by IL pore blockage.

**Figure 6:**
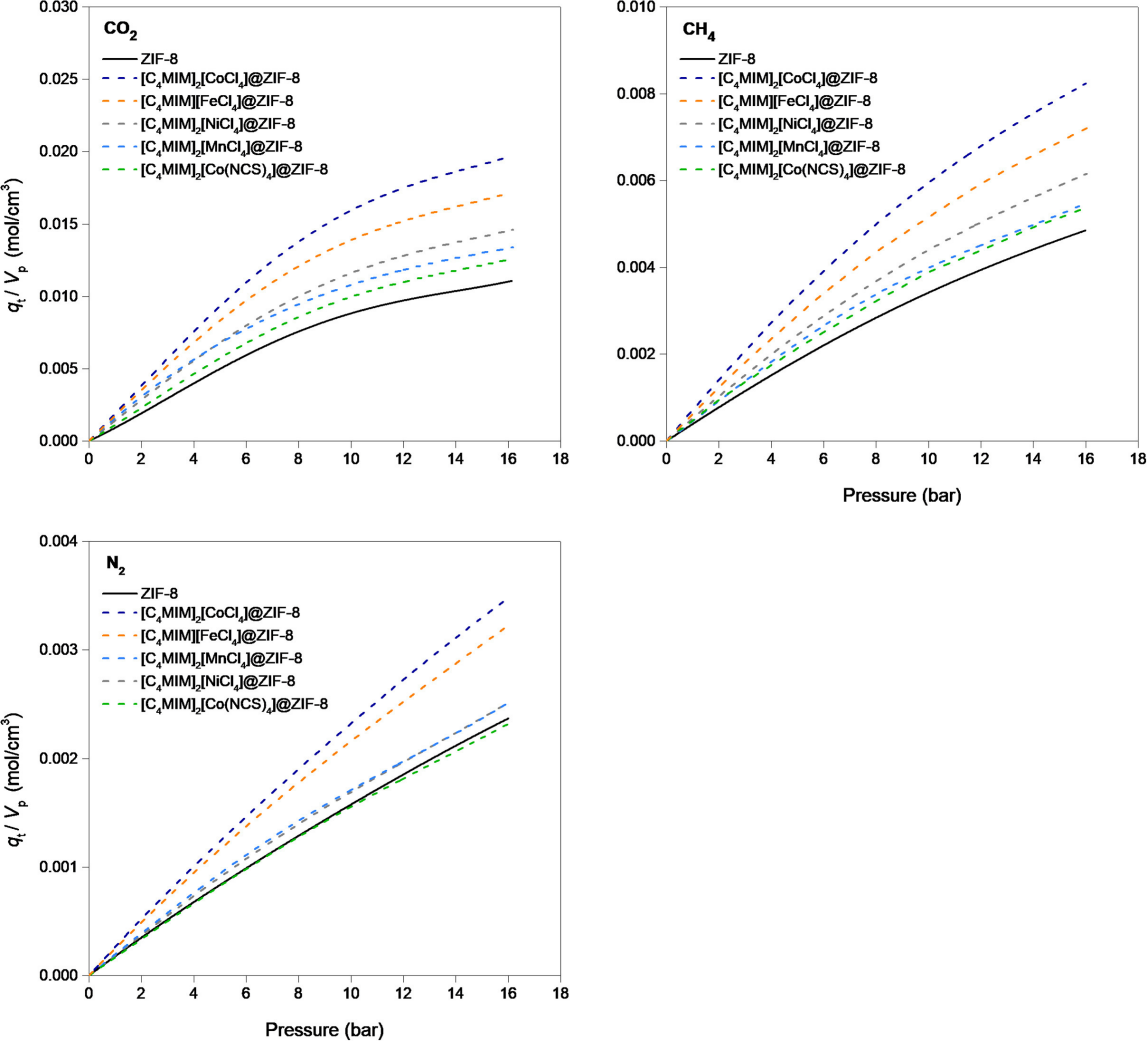
Single-component adsorption–desorption equilibrium isotherms of CO_2_, CH_4_, and N_2_ in pristine ZIF-8 and the magIL@ZIF-8 composites at 303 K. The data are shown in adsorbed moles of gas per cubic centimeter of pore volume of the respective material (obtained by the N_2_ adsorption–desorption equilibrium data at 77 K).

**Figure 7:**
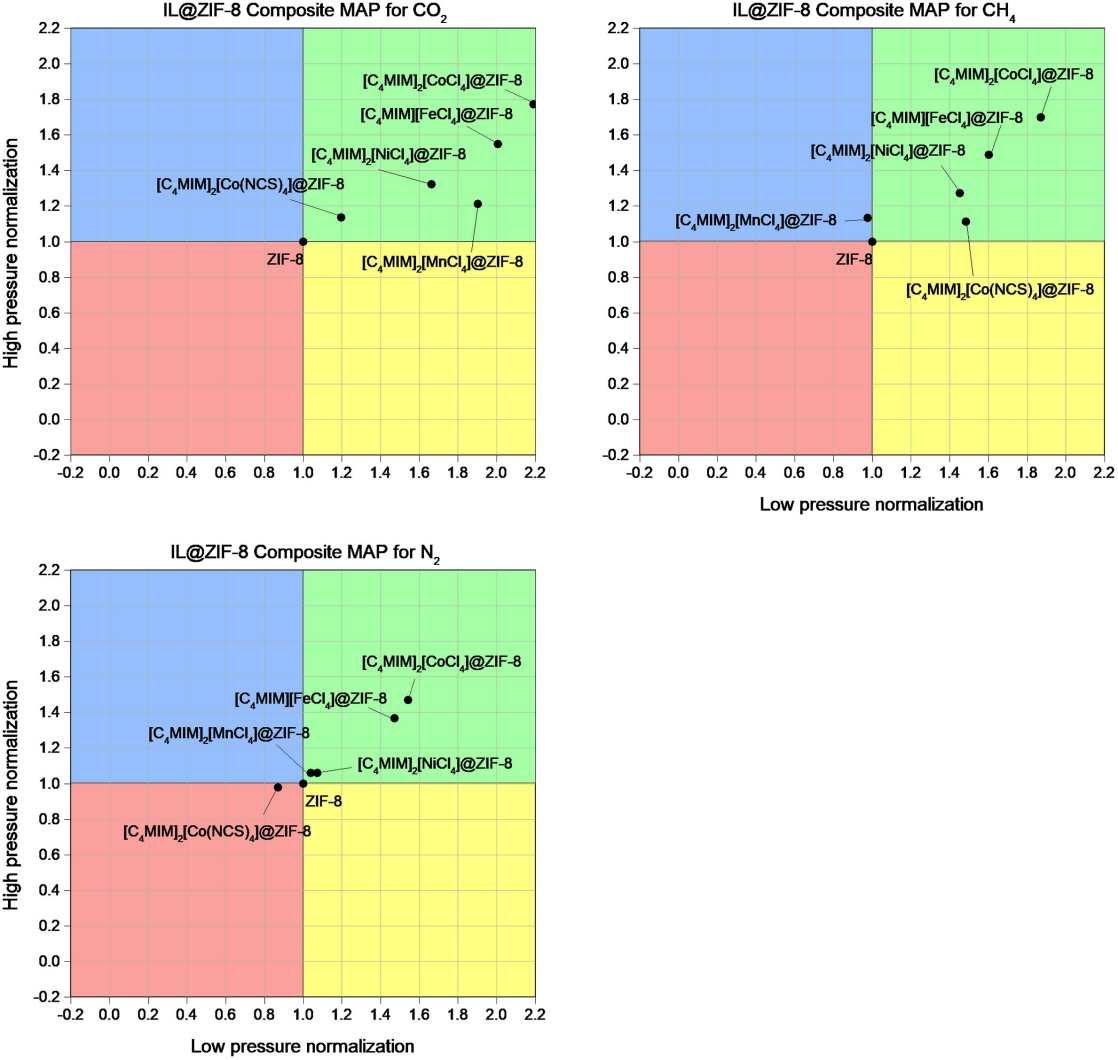
MAPs of magIL@ZIF-8 for the adsorption of CO_2_, CH_4_, and N_2_ at 303 K. ZIF-8 is the normalization reference considered in the MAP calculation for both low- (0.5 bar) and high-pressure (16 bar) normalizations.

Another approach to analyze the adsorption data collected in this work is to evaluate the gas uptake of the magIL@ZIF-8 composites per volume of available pores, as shown in Figure 6. Herein, the adsorption capacity is attained dividing the gas uptake of the adsorbent at a given pressure value by the measured total pore volume. Thus, a total gas uptake (*q*_t_/*V*_p_) is obtained and expressed in mole of gas per cm^3^ of available pore volume of the solid. It is observed that composite materials generally present more gas uptake per available pore volume, and that the choice of coordination center along with the ligand is, once again, a critical point when designing these materials. Total gas uptake expressed in *q*_t_/*V*_p_ of the composites was then normalized using the *q*_t_/*V*_p_ of ZIF-8 as the reference

(4) $$\begin{array}{r} {\left(\frac{q_{\text{t}}}{V_{\text{p}}}\right)}_{\text{normalized}}=\frac{{\left(\frac{q_{\text{t}}}{V_{\text{p}}}\right)}_{\text{composite}}}{{\left(\frac{q_{\text{t}}}{V_{\text{p}}}\right)}_{\text{ZIF}-8}} \end{array}$$

for two distinct pressures (0.5 and 16 bar, see Supplementary Tables 7–9). Figure 7 maps the normalized data for high- and low-pressure ranges. The results obtained for CO_2_ adsorption indicate that these magIL@ZIF-8 composites adsorb more gas per available total pore volume than the pristine ZIF-8 for the whole pressure range considered in this work (green MAP quadrant). The same happens for CH_4_, with [C_4_MIM]_2_[MnCl_4_]@ZIF-8 being the exception as it shows a slightly superior normalized *q*_t_/*V*_p_ at high pressure (blue MAP quadrant). As for N_2_, it follows the trend of the other two gases with [C_4_MIM]_2_[Co(NCS)_4_]@ZIF-8 showing a negative IL incorporation effect in all pressure ranges (red MAP quadrant). In a previous study (Ferreira et al., 2019), in particular for CO_2_, some IL@ZIF-8 materials were only suitable for low pressure (yellow MAP quadrant). The composite [C_2_MIM][NTf_2_]@ZIF-8 showed potential but never surpassed the normalized value of 1.2 in both pressure ranges. In this work, the produced magIL@ZIF-8 can surpass the normalized value of 1.8 for high- and low-pressure ranges, showing in particular more potential for high pressure applications. This is due to their normalized *q*_t_/*V*_p_ values that largely surpass the ones obtained in the previous study. It is important to stress that this approach also indicates a specific consequence of IL impregnation. As an example, while the [C_4_MIM]_2_[CoCl_4_]@ZIF-8 composite shows a pore loss of more than 65%, its adsorption capacity at 1 bar is only reduced in about 30%. This derives from the superior adsorption per cm^3^ of the available pore volume of this composite that is *circa* 80% superior to ZIF-8. From the results, we confirm that the presence of the IL in the MOF structure clearly affects the adsorption performance of these materials. If on one hand the pore volume loss affects negatively the adsorption capacity, on the other hand, the enhancement of the adsorption capacity per available pore volume has the opposite effect and partially cancels the pore volume loss effect.

The adsorption-desorption pure component isotherms were fitted using the Toth adsorption isotherm model (Do, 1998)

(5) $$\begin{array}{r} q(P)=q_{\text{sat}}\frac{bP}{{\left[1+{\left(bP\right)}^{t}\right]}^{1/t}} \end{array}$$

where *q*_sat_ is the saturation adsorbed quantity, *b* is the affinity constant and *t* is the system heterogeneity constant.

The ideal CO_2_/CH_4_ and CO_2_/N_2_ selectivities were determined by

(6) $$\begin{array}{r} S_{\text{C}{\text{O}}_{2}/\text{C}{\text{H}}_{4}}=\frac{q_{\text{t C}{\text{O}}_{2}}}{q_{\text{t C}{\text{H}}_{4}}} \end{array}$$

(7) $$\begin{array}{r} S_{\text{C}{\text{O}}_{2}/{\text{N}}_{2}}=\frac{q_{\text{t C}{\text{O}}_{2}}}{q_{\text{t }{\text{N}}_{2}}} \end{array}$$

considering the partial pressure of the gases in the mixtures. The obtained parameters are presented in Supplementary Tables 10–12.

The calculated parameters describe the adsorbent-adsorbate systems. For all gases, the maximum adsorbed amount (*q*_s_) of ZIF-8 is always superior to those of the composites (exception made for [C_4_MIM][FeCl_4_]@ZIF-8 for N_2_). This was expected since, at high pressures, the original MOF adsorbs more than the IL@ZIF-8 materials. The affinity parameter *b* is indicative of how strong an adsorbate molecule is attracted to the solid surface. The observed trend is that the gas that is most adsorbed, CO_2_, has the highest *b* values while N_2_ shows the lowest ones, as was expected. Nevertheless, given that the values are close zero, all gases are weakly attracted to the surface of the materials studied herein. The *t* parameter is the heterogeneity parameter and deviation from the unity indicates a higher degree of heterogeneity of the system. This seems to be the case for CO_2_, while for the other gases the systems are essentially homogeneous.

**Figure 8:**
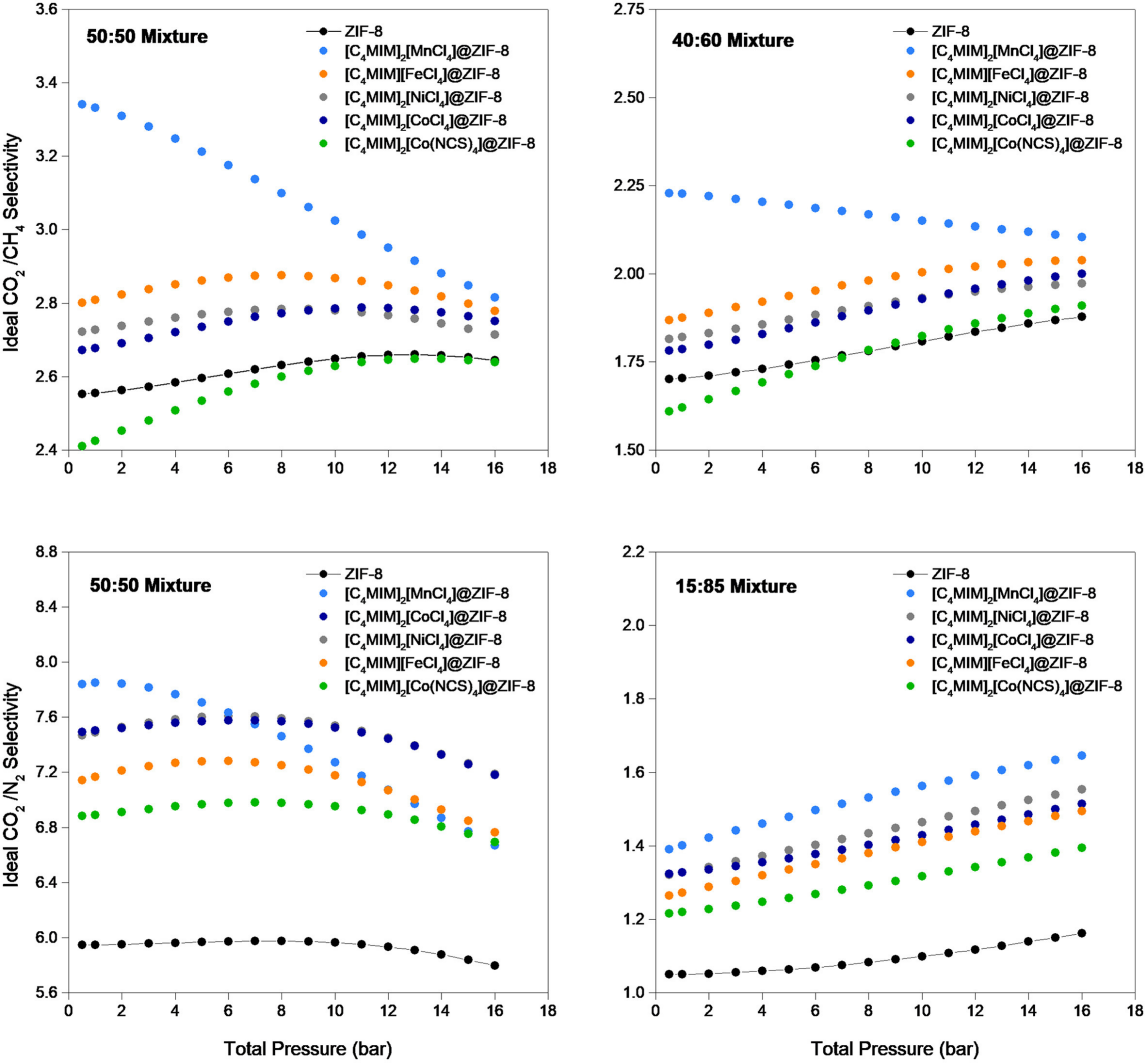
Ideal CO_2_/CH_4_ and CO_2_/N_2_ selectivities for the pristine ZIF-8 and magIL@ZIF-8 composites as a function of total pressure, determined from the Toth isotherm model fitting of the single-component adsorption-desorption isotherms measured at 303 K and in the pressure range of 0.5-16 bar.

In Figure 8, the calculated ideal CO_2_/CH_4_ and CO_2_/N_2_ selectivities are presented. It can be observed that the ILs incorporation usually leads to more selective materials, as all composites show superior ideal CO_2_/N_2_ selectivity than the one obtained in ZIF-8, and only [C_4_MIM]_2_[Co(NCS)_4_]@ZIF-8 is less selective than the original MOF when considering CO_2_/CH_4_ mixtures. For bulk CO_2_/CH_4_ and CO_2_/N_2_ mixtures such as 40:60 and 15:85 wt.%, respectively, it seems that most composites become increasingly selective with the increase of pressure. This trend was not expected, as usually IL impregnation leads to composites that are selective at low pressures, but their selectivity performance is unfavored at high pressures (Mohamedali et al., 2018; Ferreira et al., 2019). If biogas is considered, a 40:60 wt.% CO_2_/CH_4_ mixture at 10 bar of total pressure, selectivity increases from 1 to 19 %. When changing the application to CO_2_ separation from flue gas, a 15:85 wt.% CO_2_/N_2_ mixture at 1 bar of total pressure, the composites show an increase between 16 and 33 % in selectivity performance. [C_4_MIM]_2_[MnCl_4_]@ZIF-8 is the most selective composite in the lower to moderate pressure range, for all the mixtures considered, and the one that adsorbs less gas. This tendency has already been reported for other IL@MOF materials (Ferreira et al., 2019).

## Conclusions

This study reports the production of several novel composite materials consisting of magnetic ILs and ZIF-8 to evaluate the adsorption capacity and selectivity performance, targeting gas separation applications.

Characterization results indicate the success of the IL impregnation without compromising the ZIF-8 crystalline structure, and, by extension, all composites are also crystalline materials. It was observed that magIL-MOF interactions may directly affect the thermal stability of the composite, when compared with its neat IL. The magIL@ZIF-8 materials also showed significant total pore volume and specific surface area losses due to IL incorporation into ZIF-8.

CO_2_, CH_4_, and N_2_ adsorption-desorption equilibrium isotherms showed that, in both low- and high-pressure regimes, no magIL@ZIF-8 composite could surpass the original ZIF-8 in terms of gas adsorption. This was explained by the low pore volumes of the composites due to ZIF-8 pores blockage/occupation by the IL. This indicates that the new sorption sites created by IL impregnation were not strong enough to overcome the pore volume loss. The coordination center effect was studied, where the same IL cation and same anion ligand were kept but the metal atom was changed. In terms of gas uptake, the order of best anions is [CoCl_4_]^2−^ > [NiCl_4_]^2−^ > [FeCl_4_]^−^ > [MnCl_4_]^2−^ for CO_2_ and CH_4_, while for N_2_ the order is [CoCl_4_]^2−^ > [FeCl_4_]^−^ > [NiCl_4_]^2−^ > [MnCl_4_]^2−^. Keeping the metal center that adsorbed most, the ligand effect was studied. For gas adsorption, the ligand Cl performed better than NCS.

Two distinct approaches were considered for the adsorption data analysis: the use of an IL-free basis and calculations of adsorbed amounts per available pore volume. With the first one, it was concluded that the nature and structure of the magILs affect differently the adsorption performance of the produced composites. From the pore volume perspective, it was observed that these magILs significantly improve the gas uptake per cm^3^ of available pore volume on the composite materials, when compared with the original ZIF-8. This happens in both low and high pressures, which was rather unexpected.

The Toth adsorption isotherm model used to fit the collected adsorption-desorption data and to calculate ideal CO_2_/CH_4_ and CO_2_/N_2_ selectivities under biogas and flue gas compositions showed interesting improvements in selectivity. The [C_4_MIM]_2_[MnCl_4_]@ZIF-8 composite presented around 20% increase for biogas conditions and over 30% increase for flue gas conditions.

## Data Availability Statement

The original contributions presented in the study are included in the article/Supplementary Materials, further inquiries can be directed to the corresponding author/s.

## Author Contributions

TF: writing—original draft preparation, writing—review and editing, visualization, methodology, formal analysis, validation, and investigation. AV, BM, and MT: validation and investigation. LE: investigation, writing—review and editing, and visualization. IE and JE: supervision, project administration, funding acquisition, conceptualization, methodology, investigation, writing—review and editing, and visualization. All authors contributed to the article and approved the submitted version.

## Conflict of Interest

The authors declare that the research was conducted in the absence of any commercial or financial relationships that could be construed as a potential conflict of interest.

## Supplementary Material

### Supporting Information Content

TGA data (Supplementary Figure 1), N_2_ adsorption–desorption isotherms of ZIF-8 and magIL@ZIF-8 composites at 77 K (Supplementary Figures 2–3), pore-size distribution of ZIF-8 and magIL@ZIF-8 composites (Supplementary Figure 4), PXRD data (Supplementary Figure 5), FT-IR spectra of ZIF-8, pure ILs, and magIL@ZIF-8 composites (Supplementary Figures 6–10), SEM and TEM imaging of magIL@ZIF-8 composites (Supplementary Figures 11–15), characterization analyses of “ethanol-washed ZIF-8” (Supplementary Figures 16–18), CO_2_ and CH_4_ net adsorption–desorption data of ZIF-8 and “ethanol-washed ZIF-8” (Supplementary Figure 19), CO_2_, CH_4_, and N_2_ total adsorption–desorption data of ZIF-8 and magIL@ZIF-8 composites at 303 K (Supplementary Tables 1–6), single-component CO_2_, CH_4_, and N_2_ adsorption–desorption ZIF-8 and magIL@ZIF-8 composite isotherms (Supplementary Figure 20), CO_2_, CH_4_, and N_2_ total adsorption uptake at 0.5 and 16 bar of pressure per cm^3^ of available pore volume and normalized *q*_t_/*V*_p_ for ZIF-8 and magIL@ZIF-8 composites (Supplementary Tables 7–9), CO_2_, CH_4_, and N_2_ fitting parameters obtained from the experimental adsorption–desorption equilibrium isotherms (Supplementary Tables 10–12).
