# Supplementary material for: Paramagnetic Ionic Liquid/Metal Organic Framework Composites for CO2/CH4 and CO2/N2 Separations
Source: Front Chem. 2020 Nov 16;8:590191. doi: 10.3389/fchem.2020.590191 (PMC7701274; doi:10.3389/fchem.2020.590191)
Supplement: Supplementary file 1 [file Data_Sheet_1.PDF]

Supporting Information

Paramagnetic Ionic Liquid/Metal Organic Framework Composites for CO_2/CH_4 and CO_2/N_2 Separations

T. J. Ferreira[1], A. V. Torres[1], B. A. de Moura[1], L. M. Esteves[1], M. Tariq[1], J. M. S. S. Esperança[1,*] and I. A. A. C. Esteves[1,*]

[1]LAQV/REQUIMTE, Faculdade de Ciências e Tecnologia, Universidade NOVA de Lisboa (FCT NOVA), Departamento de Química, Caparica, Portugal

*** Correspondence:**
jmesp@fct.unl.pt (J.M.S.S.E); iaesteves@fct.unl.pt (I.A.A.C.E.)

List of Contents

S1. Thermogravimetric Analyses (TGA) Data S-3

S2. N_2 Adsorption-Desorption Equilibrium Data at 77 K S-4

S3. Powder X-Ray Diffraction (PXRD) Data S-7

S4. Fourier Transformed Infrared (FT-IR) Spectroscopy Data S-8

S5. Scanning and Transmission Electron Microscopy (STEM) Imaging S-11

S6. Characterization Analyses of “Ethanol-washed ZIF-8” S-16

S7. Ethanol Effect on magIL@ZIF-8 Materials Preparation S-17

S8. Single-Component Adsorption-Desorption of ZIF-8 and magIL@ZIF-8 Data S-18

S9. Single-Component Adsorption-Desorption of ZIF-8 and magIL@ZIF-8 Isotherms S-24

S10. ZIF-8 and magIL@ZIF-8 Composites Uptake per Available Pore Volume and Normalized Values S-25

S11. CO_2, CH_4 and N_2 Fitting Parameters for ZIF-8 and magIL@ZIF-8 Isotherms S-27

S1. Thermogravimetric Analyses (TGA) Data

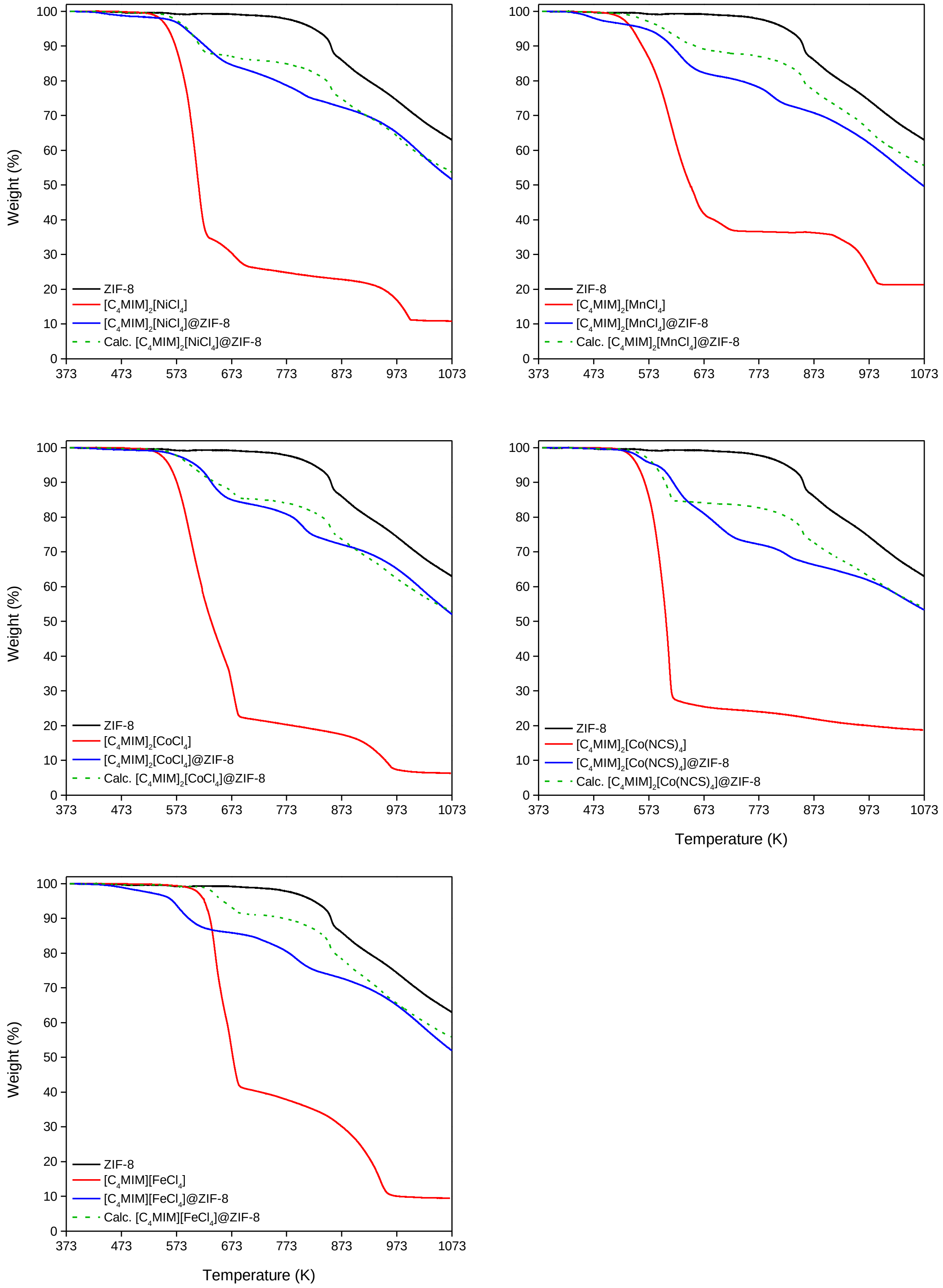

Figure S1. Obtained TGA thermograms of pristine ZIF-8, neat ILs and magIL@ZIF-8 composites (experimental and calculated).

S2. N_2 Adsorption-Desorption Equilibrium Data at 77 K

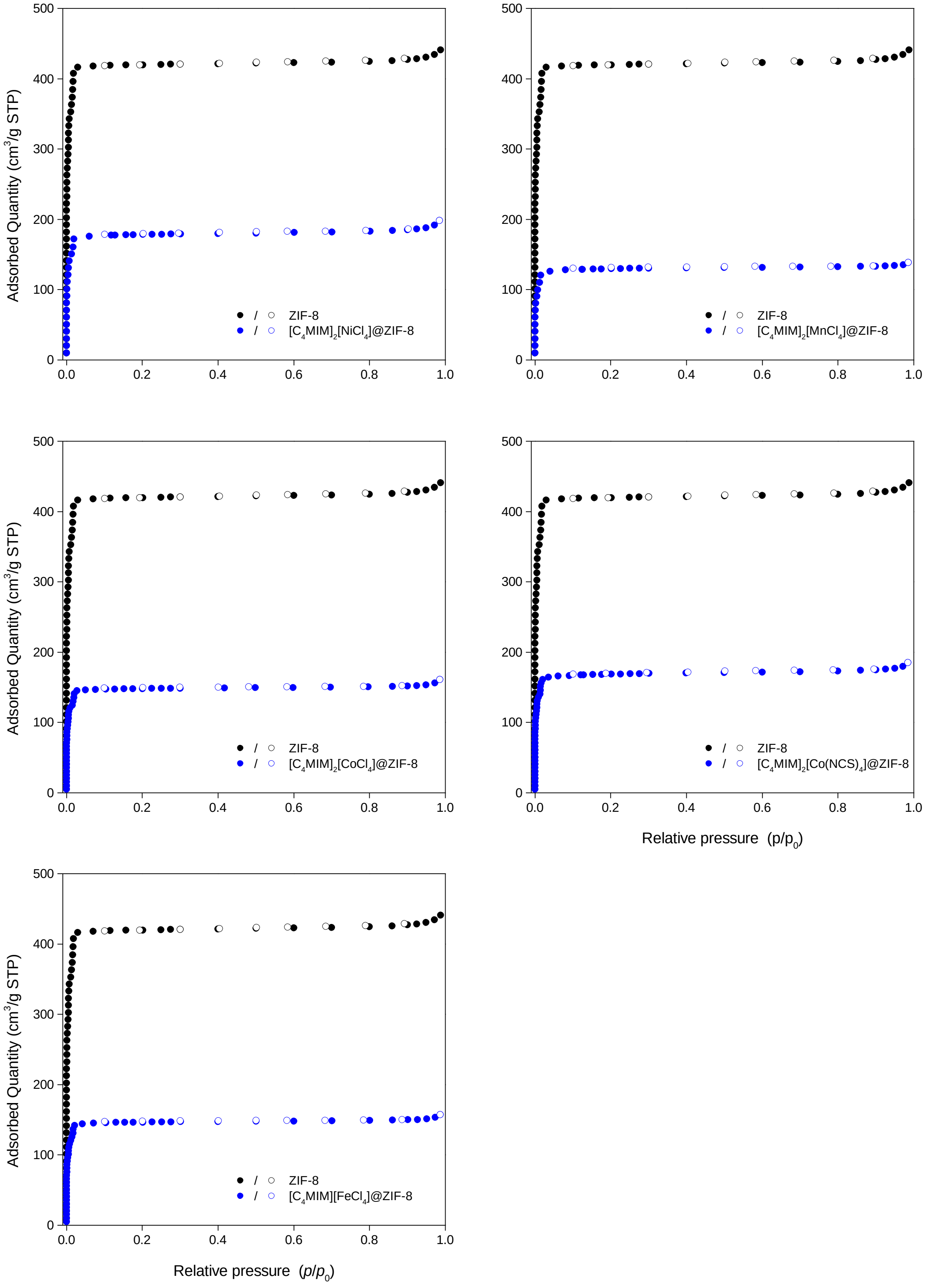

Figure S2. N_2 adsorption-desorption equilibrium isotherms at 77 K of pristine ZIF-8 and magIL@ZIF-8 composites. Closed and open symbols denote adsorption and desorption equilibria data, respectively.

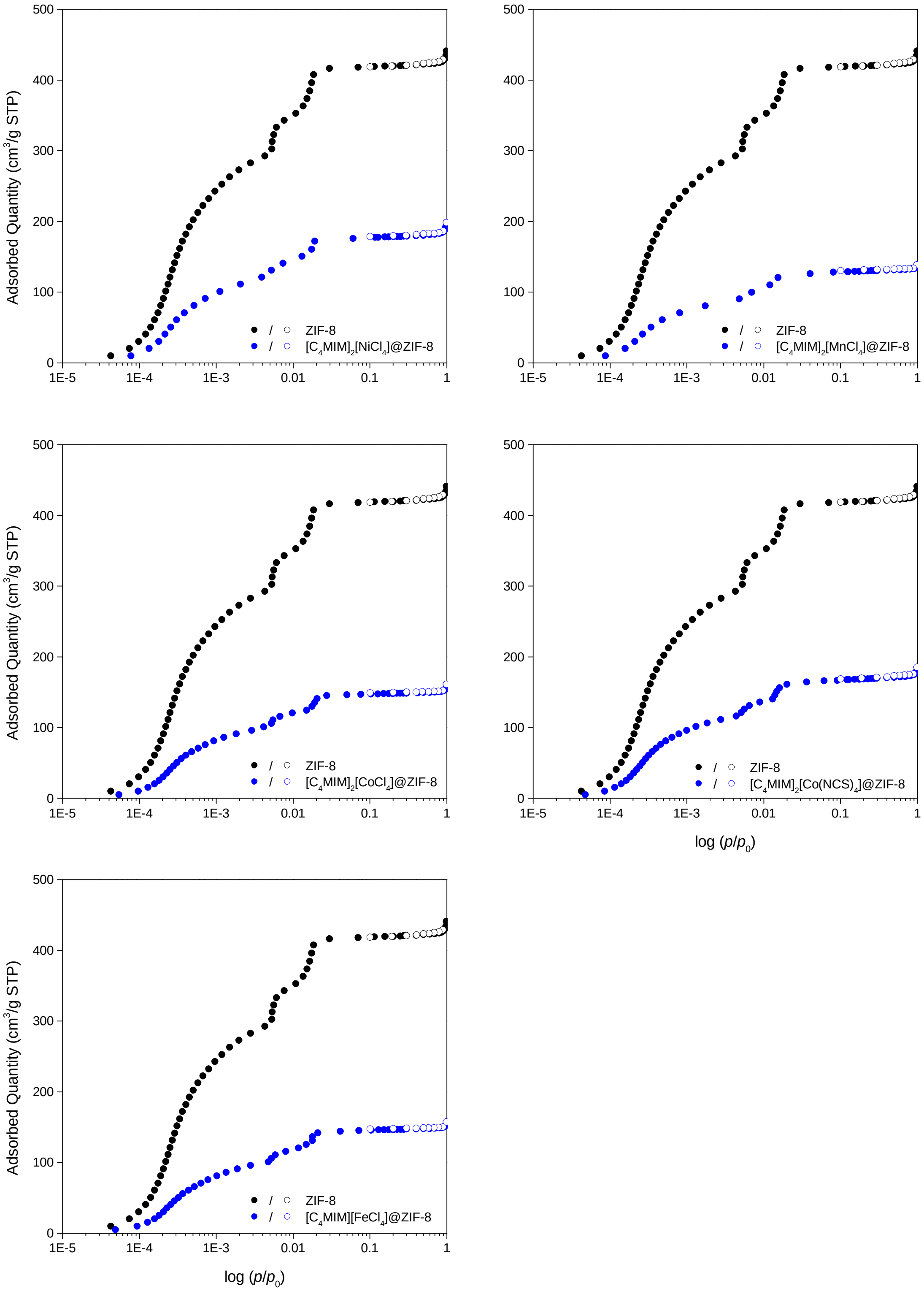

Figure S3. N_2 adsorption-desorption equilibrium isotherms at 77 K of pristine ZIF-8 and magIL@ZIF-8 composites, considering log p/p_0. Closed and open symbols denote adsorption and desorption equilibria data, respectively.

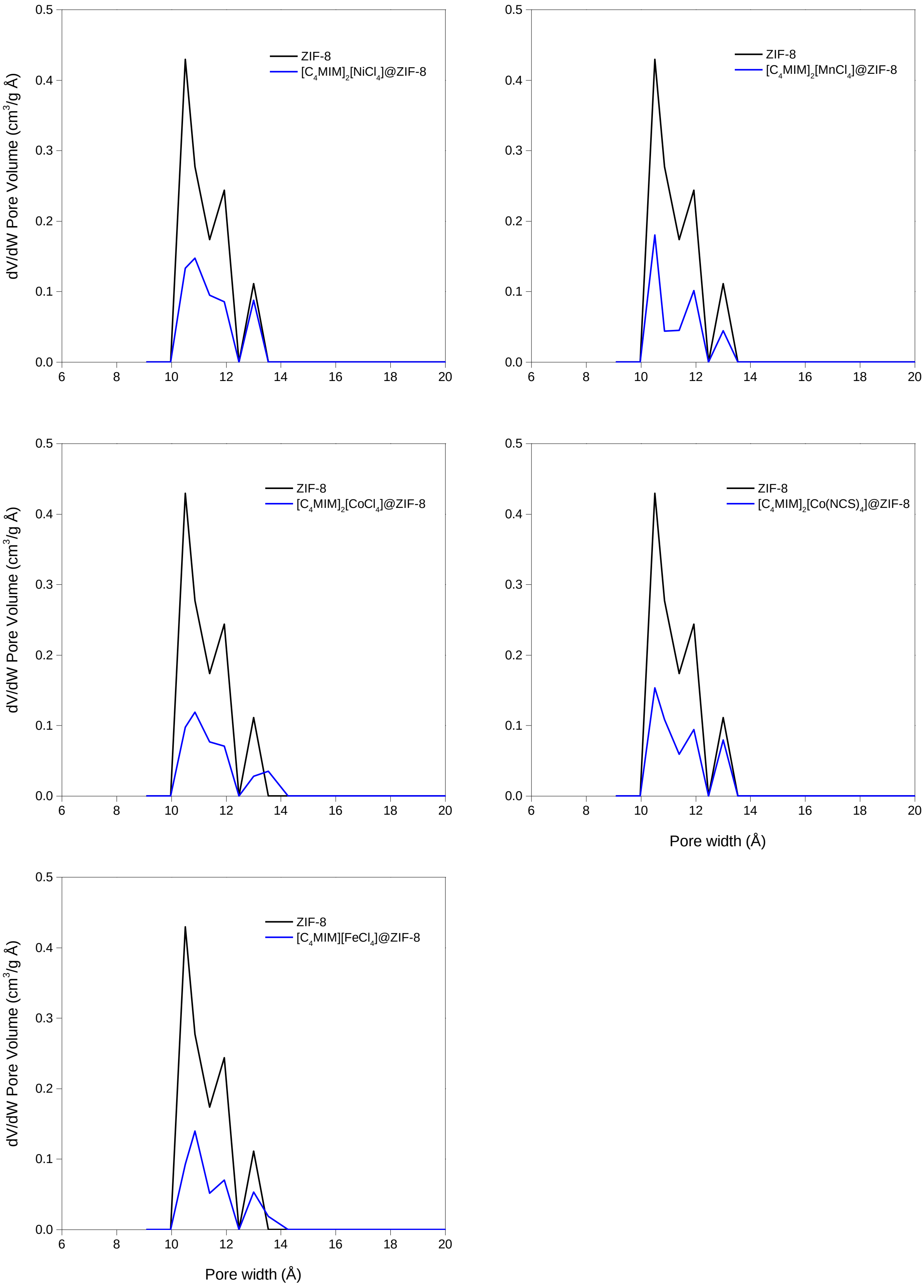

Figure S4. Pore size distribution of pristine ZIF-8 and magIL@ZIF-8 composites.

S3. Powder X-Ray Diffraction (PXRD) Data

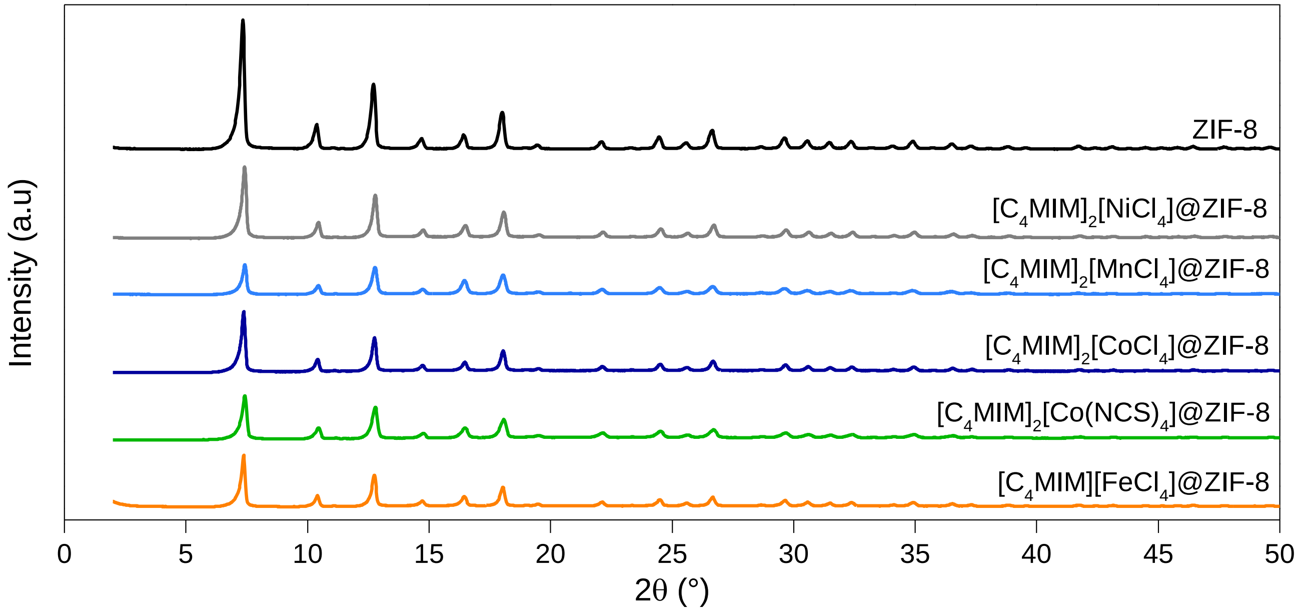

Figure S5. PXRD diffractograms of pristine ZIF-8 and magIL@ZIF-8 composites.

S4. Fourier Transformed Infrared (FT-IR) Spectroscopy Data

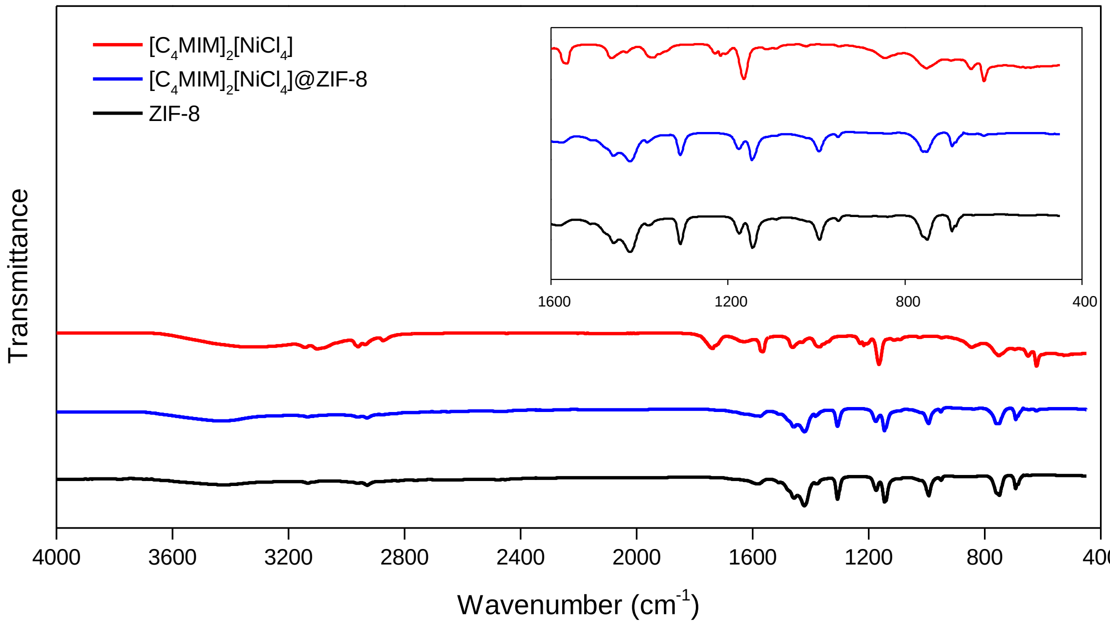

Figure S6. FT-IR spectra of pristine ZIF-8, pure $[C_4MIM]_2[NiCl_4]$, and $[C_4MIM]_2[NiCl_4]$@ZIF-8 composite. The inset details the low wavenumber range.

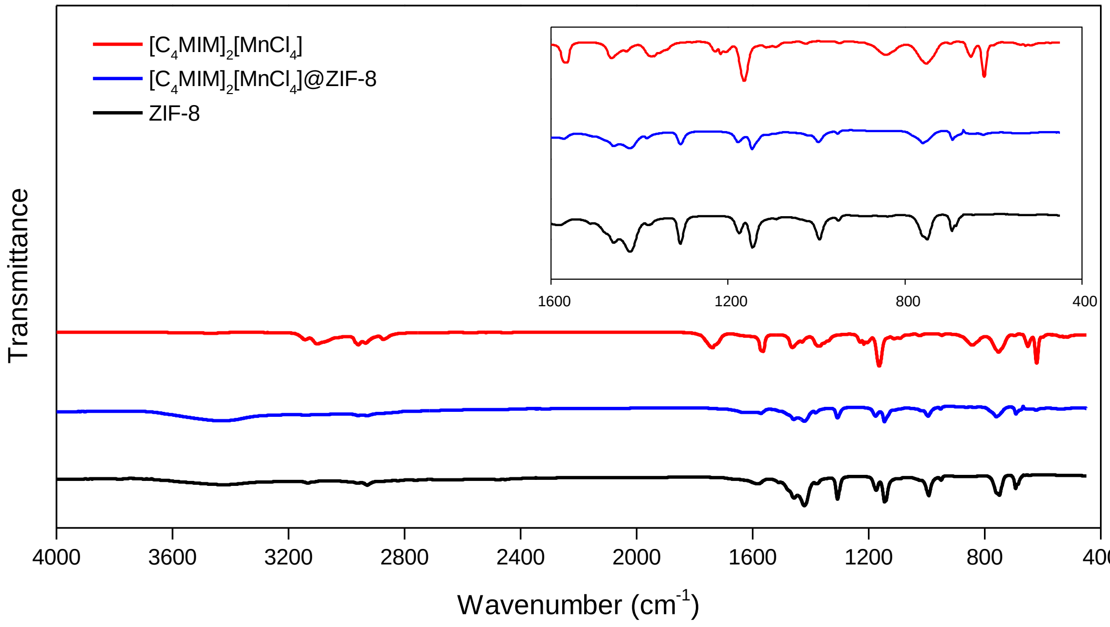

Figure S7. FT-IR spectra of pristine ZIF-8, pure $[C_4MIM]_2[MnCl_4]$, and $[C_4MIM]_2[MnCl_4]$@ZIF-8 composite. The inset details the low wavenumber range.

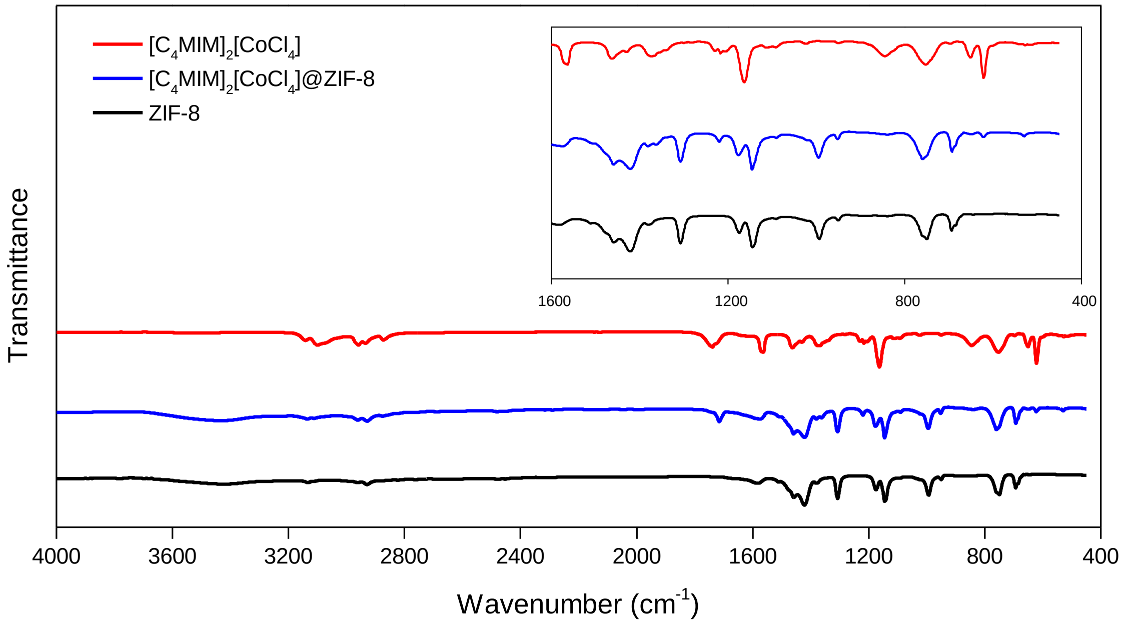

Figure S8. FT-IR spectra of pristine ZIF-8, pure $[C_4MIM]_2[CoCl_4]$, and $[C_4MIM]_2[CoCl_4]$@ZIF-8 composite. The inset details the low wavenumber range.

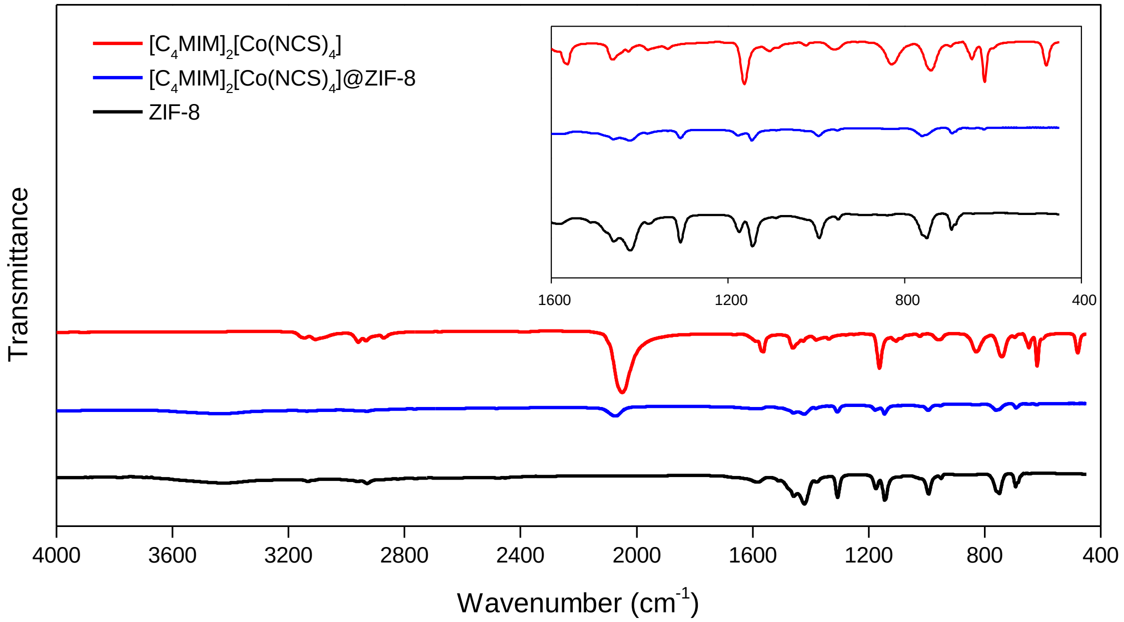

Figure S9. FT-IR spectra of pristine ZIF-8, pure $[C_4MIM]_2[Co(NCS)_4]$, and $[C_4MIM]_2[Co(NCS)_4]$@ZIF-8 composite. The inset details the low wavenumber range.

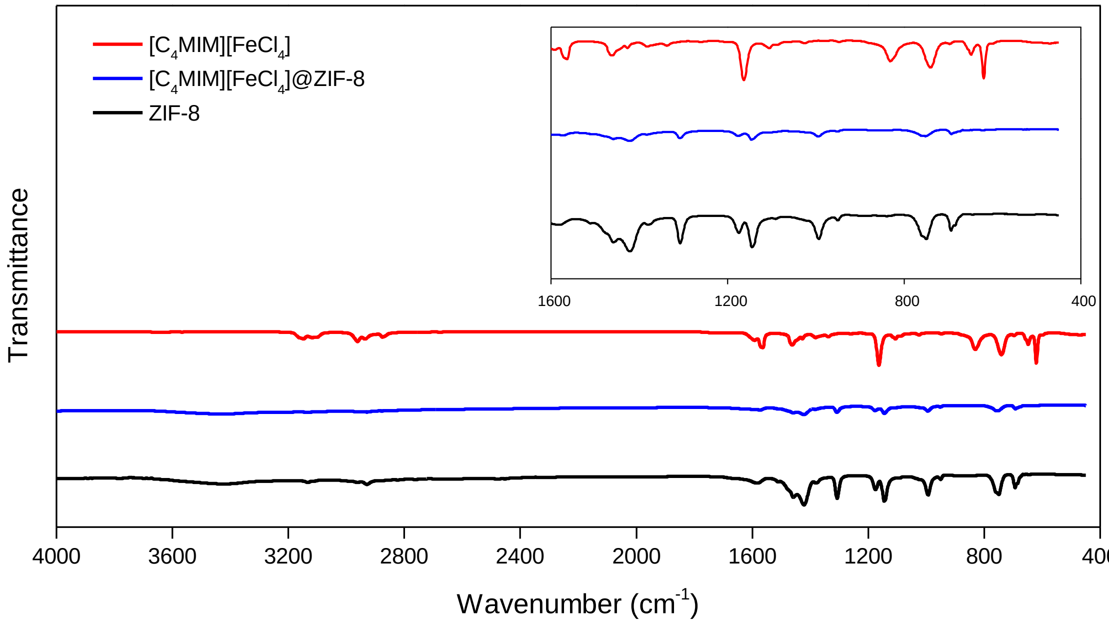

Figure S10. FT-IR spectra of pristine ZIF-8, pure [C_4MIM][$FeCl_4$], and [C_4MIM][$FeCl_4$]@ZIF-8 composite. The inset details the low wavenumber range.

S5. Scanning and Transmission Electron Microscopy (STEM) Imaging

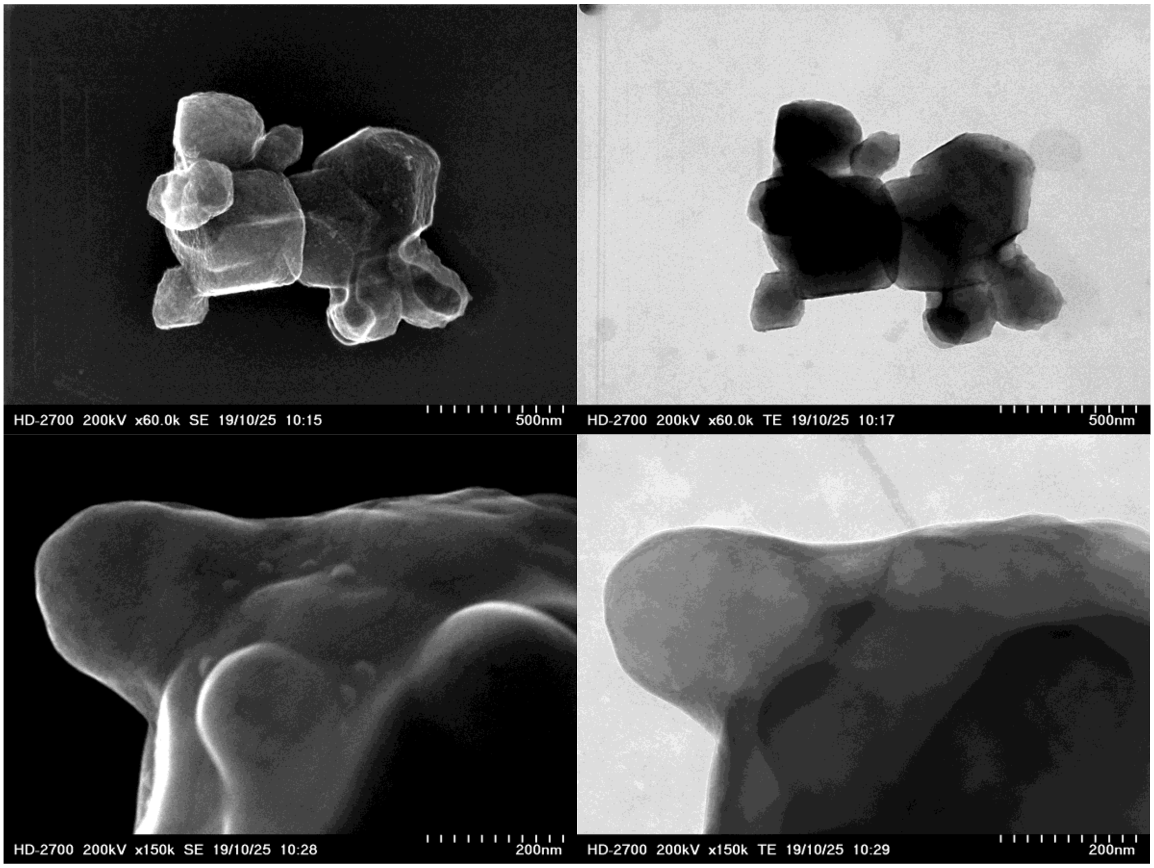

Figure S11. SEM (left) and TEM (right) micrographs of $[C_4MIM]_2[NiCl_4]$@ZIF-8 (from top to bottom: ×60k and ×150k magnifications).

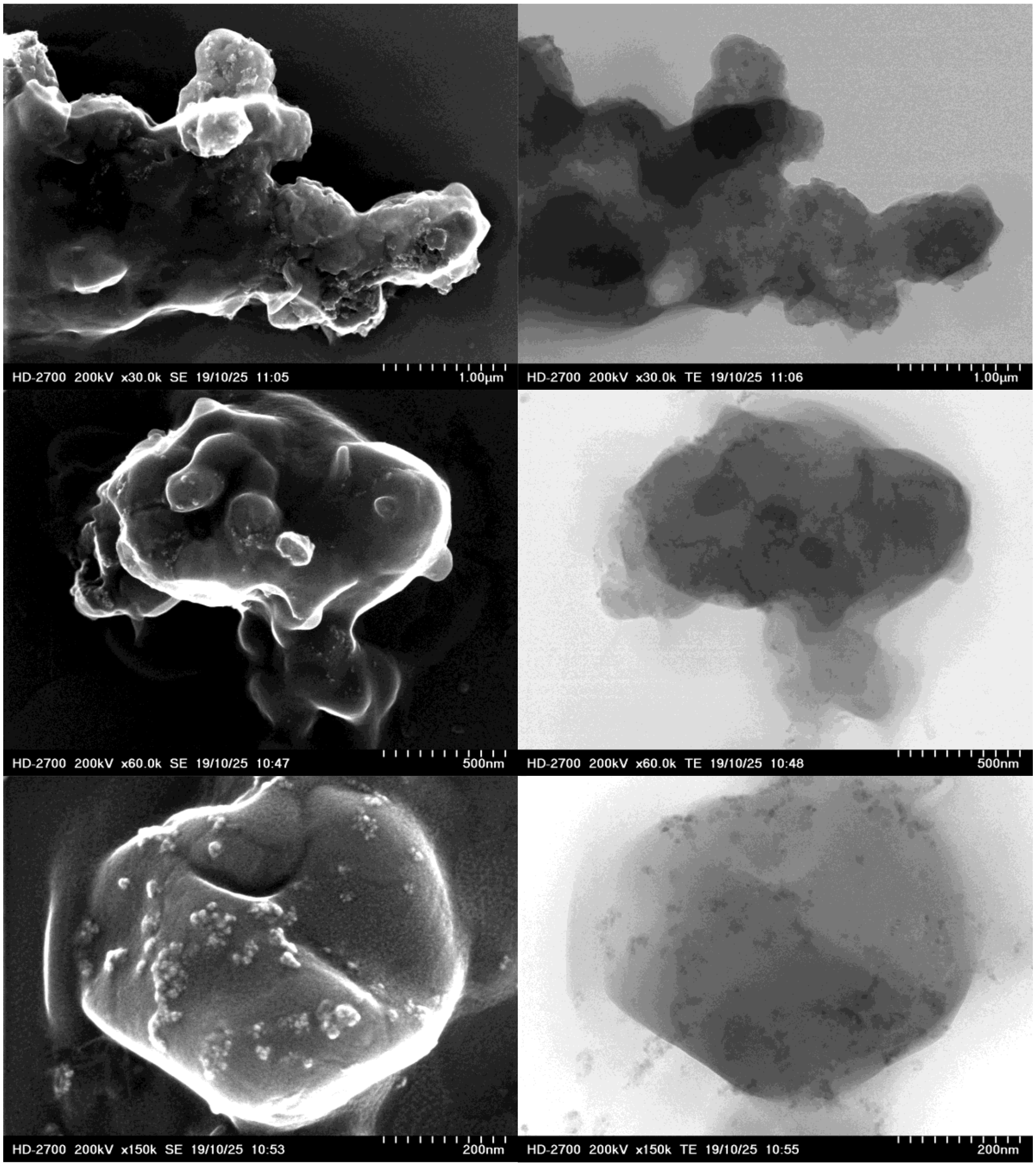

Figure S12. SEM (left) and TEM (right) micrographs of $[C_4MIM]_2[MnCl_4]$@ZIF-8 (from top to bottom: ×30k, ×60k and ×150k magnifications).

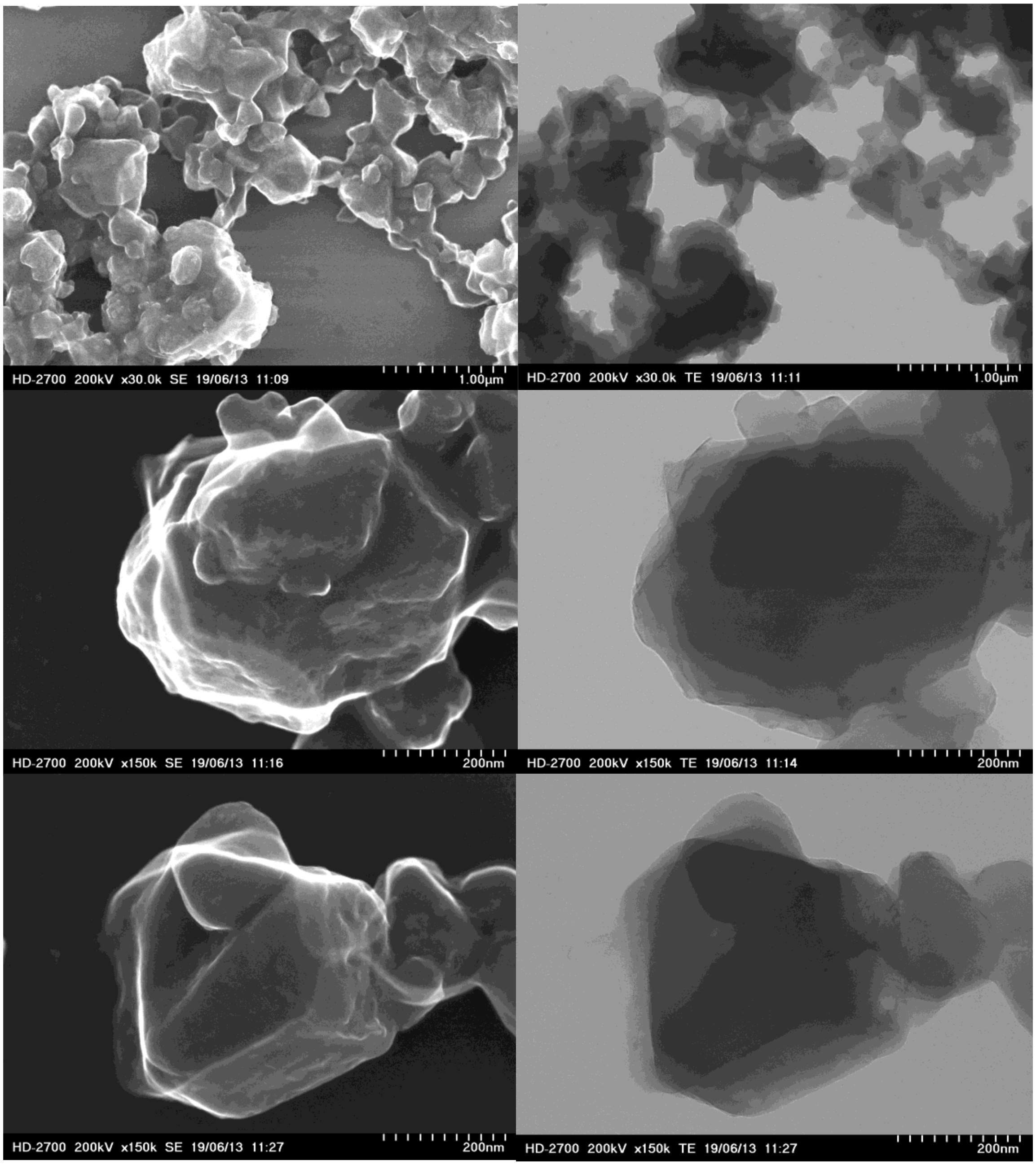

Figure S13. SEM (left) and TEM (right) micrographs of $[C_4MIM]_2[CoCl_4]$@ZIF-8 (from top to bottom: ×30k and ×150k magnifications).

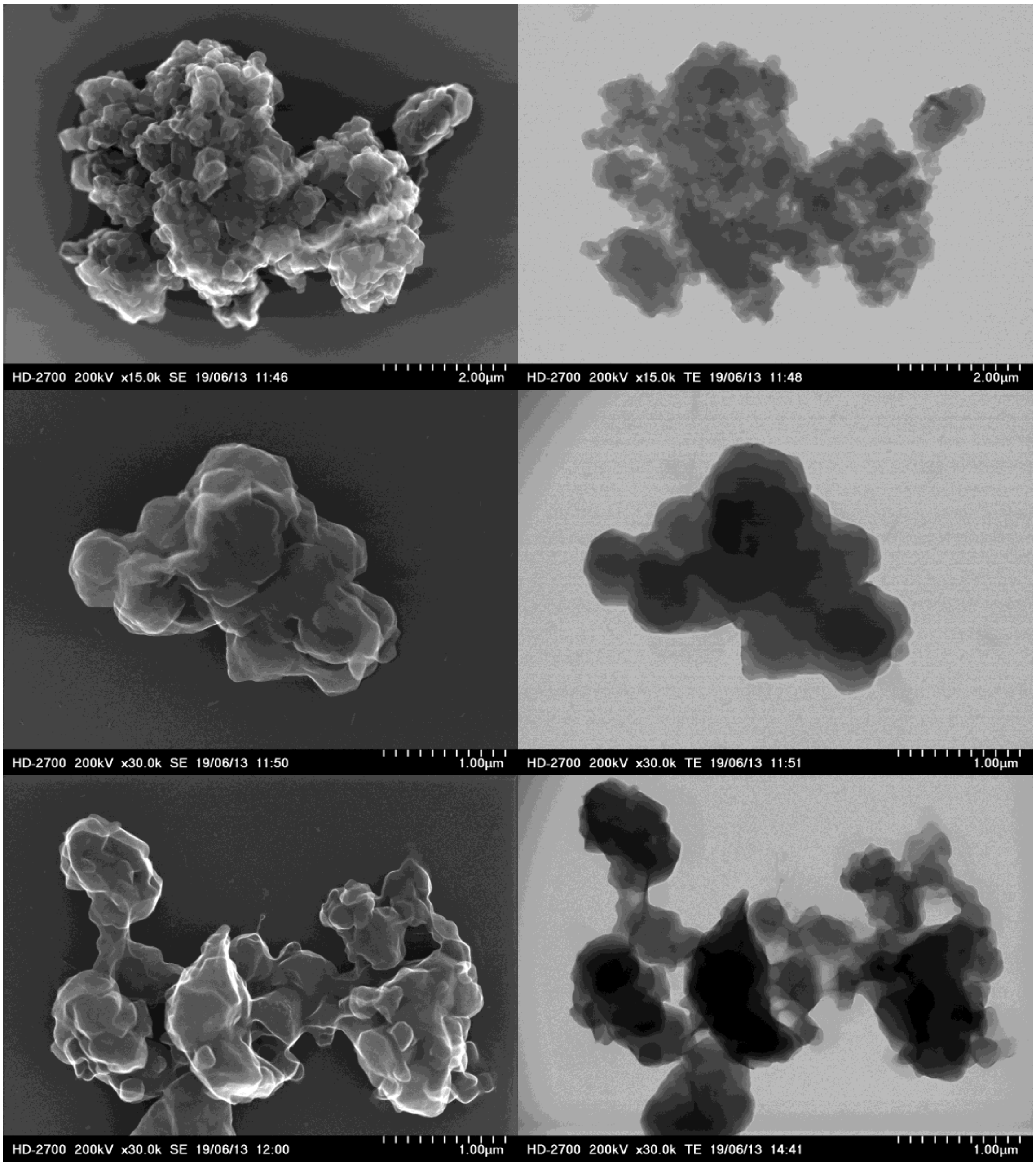

Figure S14. SEM (left) and TEM (right) micrographs of $[C_4MIM]_2[Co(NCS)_4]$@ZIF-8 (from top to bottom: ×15k and ×30k magnifications).

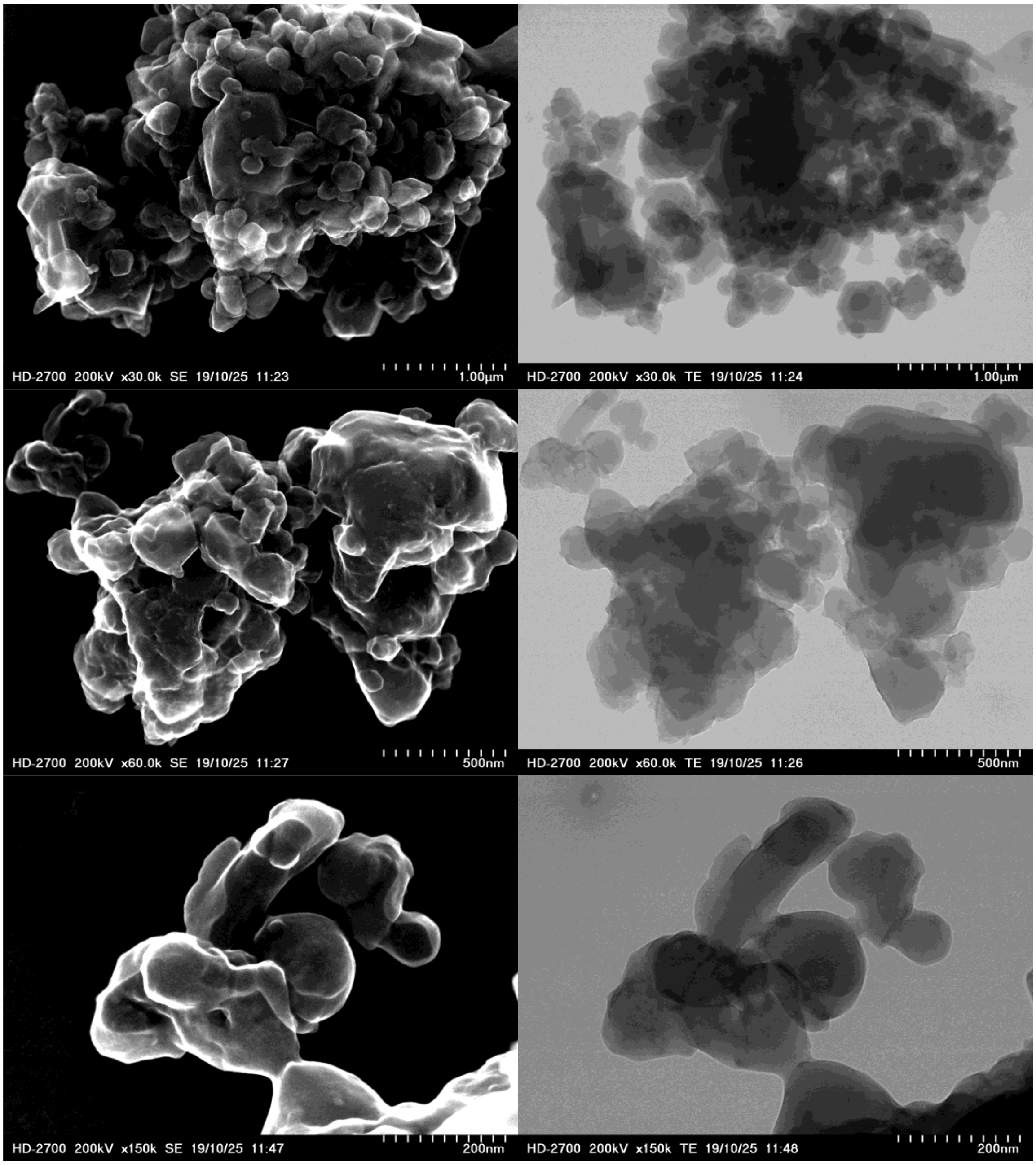

Figure S15. SEM (left) and TEM (right) micrographs of $[C_4MIM][FeCl_4]$@ZIF-8 (from top to bottom: ×30k, ×60k and ×150k magnifications).

S6. Characterization Analyses of "Ethanol-washed ZIF-8"

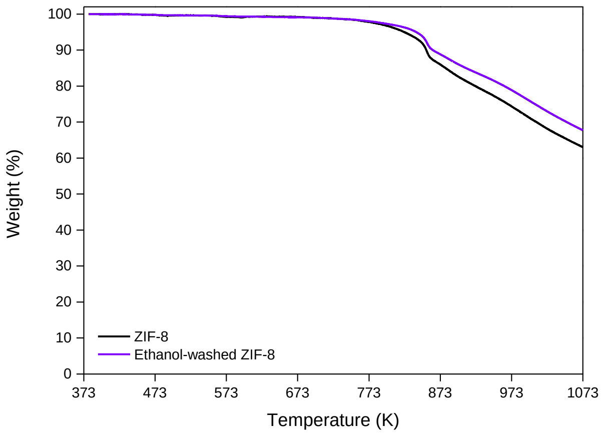

Figure S16. TGA thermograms of pristine ZIF-8 and "ethanol-washed ZIF-8".

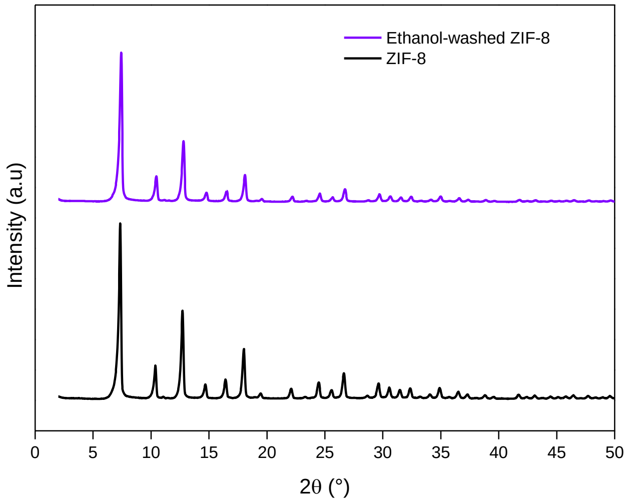

Figure S17. PXRD diffractograms of pristine ZIF-8 and "ethanol-washed ZIF-8".

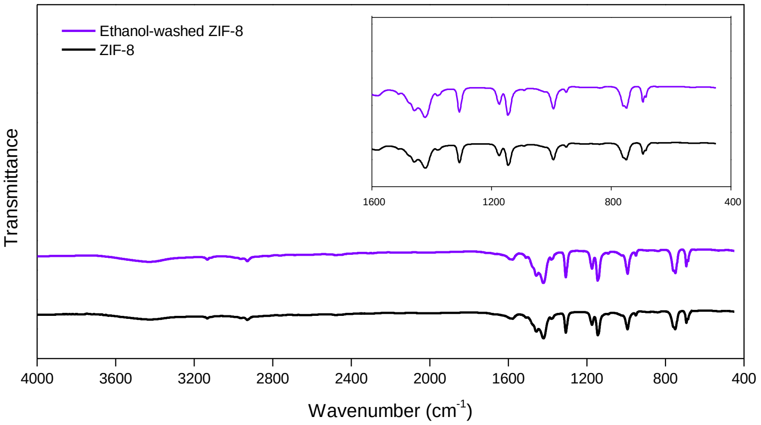

Figure S18. FT-IR spectra of pristine ZIF-8 and "ethanol-washed ZIF-8".

S7. Ethanol Effect on magIL@ZIF-8 Materials Preparation

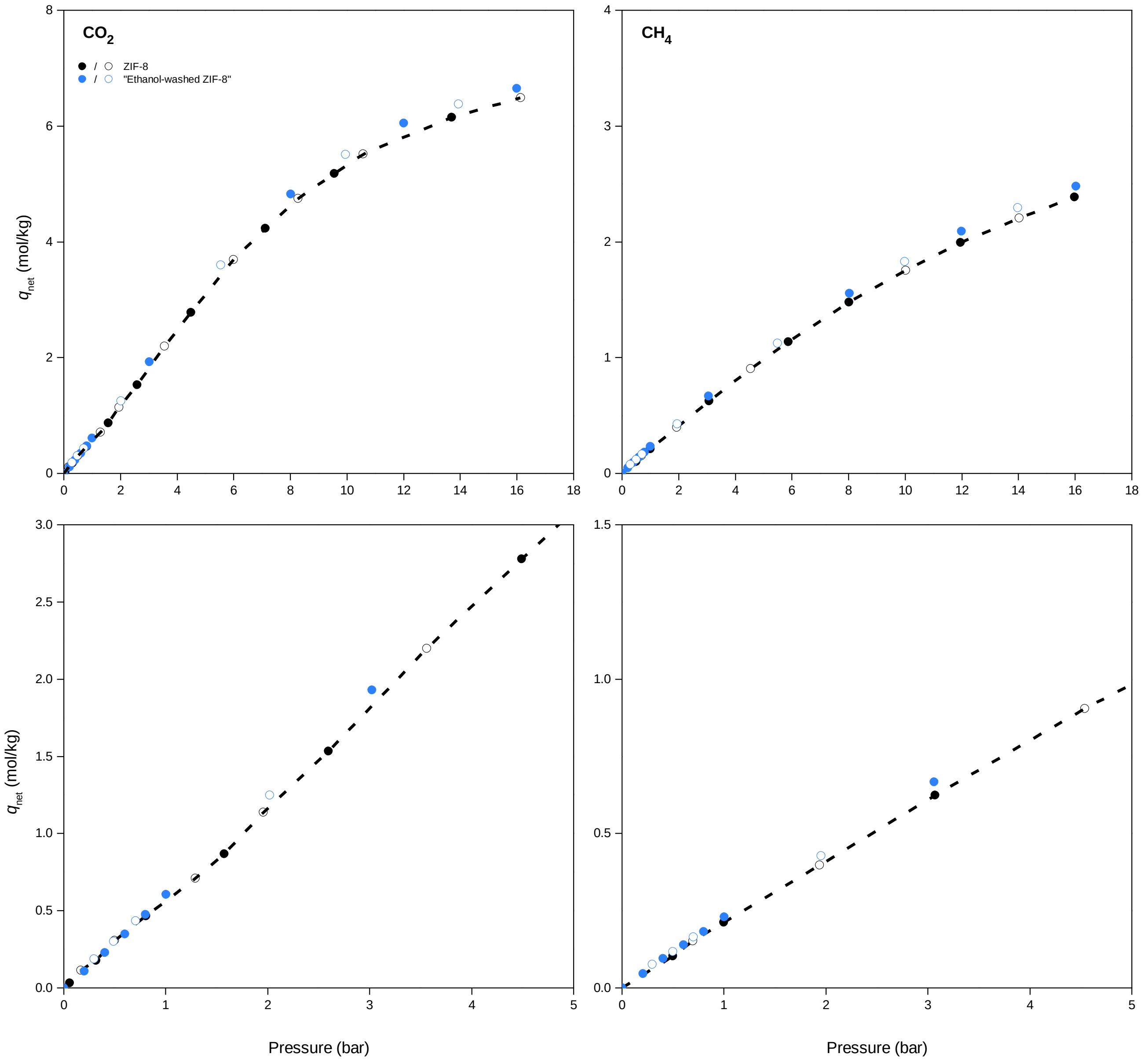

Figure S19. Effect of ethanol as washing solvent in the CO_2 and CH_4 adsorption capacities. The black dashed line is a guide-to-the-eye relative to the pristine ZIF-8.

S8. Single-Component Adsorption-Desorption of ZIF-8 and magIL@ZIF-8 Data

Table S1. CO_2, CH_4 and N_2 adsorption-desorption equilibrium data at 303 K for ZIF-8.

CO_2		CH_4		N_2	
P (bar)	q_t (mol/kg)	*P* (bar)	q_t (mol/kg)	*P* (bar)	q_t (mol/kg)
0.06	0.03	0.50	0.13	0.50	0.06
0.32	0.19	1.00	0.26	1.00	0.12
0.81	0.51	3.07	0.79	2.94	0.34
1.57	0.95	5.88	1.45	6.05	0.67
2.60	1.67	8.01	1.91	7.99	0.86
4.49	3.02	11.95	2.64	12.08	1.25
7.11	4.63	15.98	3.26	16.01	1.59
9.55	5.71	14.02	2.97	14.01	1.42
13.70	6.93	10.02	2.29	9.89	1.05
16.14	7.42	4.54	1.15	4.51	0.52
10.58	6.11	1.94	0.50	1.99	0.23
8.28	5.21	0.70	0.19	0.70	0.08
5.99	4.02				
3.56	2.39				
1.96	1.24				
1.29	0.78				
0.50	0.33				
0.17	0.12				

Table S2. CO_2, CH_4 and N_2 adsorption-desorption equilibrium data at 303 K for $[C_4MIM]_2[NiCl_4]$@ZIF-8.

CO_2		CH_4		N_2	
P (bar)	q_t (mol/kg)	*P* (bar)	q_t (mol/kg)	*P* (bar)	q_t (mol/kg)
0.40	0.16	0.41	0.06	0.20	0.01
0.81	0.32	0.80	0.13	0.40	0.02
1.00	0.40	1.00	0.16	0.61	0.04
3.25	1.31	3.04	0.45	0.80	0.05
5.97	2.35	6.33	0.89	1.01	0.06
8.02	2.96	8.02	1.10	3.05	0.17
11.99	3.76	12.11	1.50	6.01	0.32
16.20	4.32	16.06	1.83	8.06	0.42
13.99	4.09	14.27	1.68	11.97	0.57
10.10	3.49	9.96	1.30	15.96	0.74
4.47	1.87	4.49	0.66	14.00	0.67
1.98	0.84	1.97	0.30	9.77	0.50
0.70	0.33	0.70	0.12	4.38	0.23
0.50	0.25	0.50	0.08	1.95	0.11
0.20	0.12	0.20	0.03	0.70	0.04
				0.49	0.03
				0.30	0.02

Table S3. CO_2, CH_4 and N_2 adsorption-desorption equilibrium data at 303 K for $[C_4MIM]_2[MnCl_4]$@ZIF-8.

CO_2		CH_4		N_2	
P (bar)	q_t (mol/kg)	*P* (bar)	q_t (mol/kg)	*P* (bar)	q_t (mol/kg)
0.40	0.13	0.41	0.03	0.20	0.01
0.81	0.26	0.80	0.07	0.40	0.02
1.00	0.32	1.00	0.09	0.61	0.03
3.25	0.96	3.04	0.29	0.80	0.03
5.97	1.60	6.33	0.58	1.01	0.04
8.02	1.97	8.02	0.70	3.05	0.12
11.99	2.45	12.11	0.95	6.01	0.24
16.20	2.81	16.06	1.15	8.06	0.30
13.99	2.66	14.27	1.06	11.97	0.41
10.10	2.31	9.96	0.84	15.96	0.53
4.47	1.33	4.49	0.43	14.00	0.47
1.98	0.64	1.97	0.21	9.77	0.35
0.70	0.27	0.70	0.06	4.38	0.17
0.50	0.20	0.50	0.04	1.95	0.08
0.20	0.10	0.20	0.01	0.70	0.03
				0.49	0.02
				0.30	0.01

Table S4. CO_2, CH_4 and N_2 adsorption-desorption equilibrium data at 303 K for $[C_4MIM]_2[CoCl_4]$@ZIF-8.

CO_2		CH_4		N_2	
P (bar)	q_t (mol/kg)	*P* (bar)	q_t (mol/kg)	*P* (bar)	q_t (mol/kg)
0.20	0.08	0.20	0.03	0.20	0.01
0.40	0.17	0.40	0.06	0.40	0.03
0.60	0.25	0.60	0.09	0.61	0.04
0.80	0.34	0.80	0.13	0.81	0.05
1.00	0.43	1.00	0.17	1.00	0.06
3.02	1.35	3.06	0.51	3.04	0.18
8.02	3.31	8.03	1.20	6.08	0.36
12.01	4.20	11.98	1.63	8.05	0.46
15.99	4.73	16.02	1.99	12.02	0.66
13.94	4.53	13.98	1.83	16.00	0.84
9.96	3.85	9.98	1.45	13.99	0.75
5.54	2.52	5.49	0.88	9.90	0.56
2.02	0.93	1.95	0.35	4.44	0.27
0.70	0.36	0.70	0.12	1.93	0.12
0.49	0.26	0.50	0.09	0.70	0.05
0.30	0.18	0.30	0.05	0.49	0.03
				0.30	0.02

Table S5. CO_2, CH_4 and N_2 adsorption-desorption equilibrium data at 303 K for $[C_4MIM]_2[Co(NCS)_4]$@ZIF-8.

CO_2		CH_4		N_2	
P (bar)	q_t (mol/kg)	*P* (bar)	q_t (mol/kg)	*P* (bar)	q_t (mol/kg)
0.21	0.06	0.21	0.03	0.20	0.01
0.42	0.13	0.40	0.06	0.40	0.02
0.60	0.18	0.61	0.08	0.61	0.03
0.80	0.24	0.80	0.11	0.81	0.04
1.00	0.30	1.00	0.13	1.00	0.05
3.12	0.99	3.13	0.39	3.04	0.14
6.18	1.94	8.05	0.90	6.08	0.27
8.03	2.37	12.05	1.22	8.05	0.36
12.04	3.05	16.12	1.50	12.02	0.50
16.04	3.49	14.06	1.37	16.00	0.64
13.86	3.27	10.07	1.09	13.99	0.57
10.07	2.79	5.45	0.64	9.90	0.43
5.51	1.77	1.90	0.25	4.44	0.21
1.91	0.61	0.70	0.11	1.93	0.09
0.70	0.23	0.50	0.08	0.70	0.03
0.50	0.17	0.29	0.05	0.49	0.02
0.30	0.10			0.30	0.01

Table S6. CO_2, CH_4 and N_2 adsorption-desorption equilibrium data at 303 K for $[C_4MIM][FeCl_4]$@ZIF-8.

CO_2		CH_4		N_2	
P (bar)	q_t (mol/kg)	*P* (bar)	q_t (mol/kg)	*P* (bar)	q_t (mol/kg)
0.20	0.08	0.22	0.03	0.20	0.01
0.42	0.17	0.40	0.06	0.40	0.03
0.61	0.25	0.60	0.09	0.60	0.04
0.80	0.32	0.81	0.12	0.80	0.05
1.02	0.41	1.00	0.15	1.00	0.06
3.09	1.22	3.05	0.43	3.03	0.17
8.08	2.85	8.03	1.03	6.10	0.33
12.15	3.59	11.99	1.40	8.02	0.43
16.00	4.05	16.07	1.71	12.02	0.59
13.97	3.86	13.99	1.56	16.08	0.77
9.97	3.31	10.17	1.24	13.99	0.68
5.41	2.16	5.75	0.78	9.97	0.51
1.96	0.83	2.12	0.31	4.35	0.24
0.72	0.33	0.69	0.11	1.95	0.11
0.51	0.24	0.50	0.07	0.70	0.04
0.30	0.15	0.30	0.04	0.49	0.03
				0.30	0.02

S9. Single-Component Adsorption-Desorption of ZIF-8 and magIL@ZIF-8 Isotherms

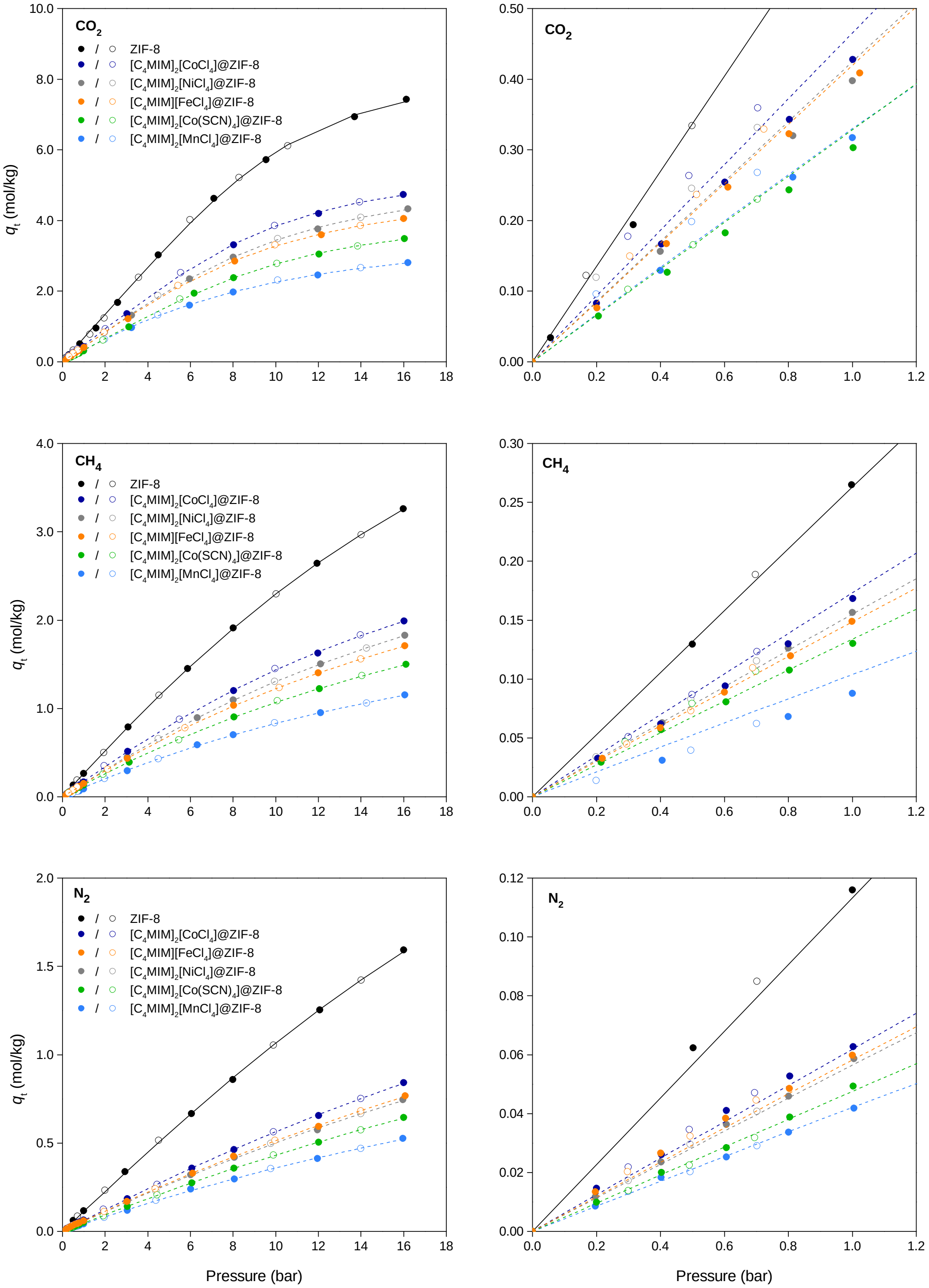

Figure S20. (Left) Single-component adsorption-desorption equilibrium isotherms of CO_2, CH_4 and N_2 in the pristine ZIF-8 and magIL@ZIF-8 composites at 303 K. Closed and open symbols denote adsorption and desorption data, respectively. Lines represent the Toth adsorption isotherm model fitting to the experimental data points. On the right side, a low-pressure range inset of the data is shown to clarify the goodness of the fittings to the experimental data.

S10. ZIF-8 and magIL@ZIF-8 Composites Uptake per Available Pore Volume and Normalized Values

Table S7. CO_2 total adsorption quantities at 16 and 0.5 bar for ZIF-8 and the produced magIL@ZIF-8 in mol/kg, mol/cm^3 of available pore volume and normalized using ZIF-8 as reference. The data is reported for 303 K.

Sample	16 bar			0.5 bar		
	q_t (mol/kg)	q_t/V_p (mol/cm^3)	Normal. q_t/V_p	q_t (mol/kg)	q_t/V_p (mol/cm^3)	Normal. q_t/V_p
ZIF-8	7.42	0.01104	1.00	0.33	0.00050	1.00
$[C_4MIM]_2[NiCl_4]$@ZIF-8	4.32	0.01459	1.32	0.25	0.00083	1.66
$[C_4MIM]_2[MnCl_4]$@ZIF-8	2.81	0.01339	1.21	0.20	0.00095	1.90
$[C_4MIM]_2[CoCl_4]$@ZIF-8	4.73	0.01957	1.77	0.26	0.00109	2.19
$[C_4MIM]_2[Co(NCS)_4]$@ZIF-8	3.49	0.01255	1.14	0.17	0.00060	1.20
$[C_4MIM][FeCl_4]$@ZIF-8	4.05	0.01708	1.55	0.24	0.00100	2.01

Table S8. CH_4 total adsorption quantities at 16 and 0.5 bar for ZIF-8 and the produced magIL@ZIF-8 in mol/kg, mol/cm^3 of available pore volume and normalized using ZIF-8 as reference. The data is reported for 303 K.

Sample	16 bar			0.5 bar		
	q_t (mol/kg)	q_t/V_p (mol/cm^3)	Normal. q_t/V_p	q_t (mol/kg)	q_t/V_p (mol/cm^3)	Normal. q_t/V_p
ZIF-8	3.26	0.00485	1.00	0.13	0.00019	1.00
$[C_4MIM]_2[NiCl_4]$@ZIF-8	1.83	0.00617	1.27	0.08	0.00028	1.45
$[C_4MIM]_2[MnCl_4]$@ZIF-8	1.15	0.00549	1.13	0.04	0.00019	0.98
$[C_4MIM]_2[CoCl_4]$@ZIF-8	1.99	0.00823	1.70	0.09	0.00036	1.87
$[C_4MIM]_2[Co(NCS)_4]$@ZIF-8	1.50	0.00539	1.11	0.08	0.00029	1.49
$[C_4MIM][FeCl_4]$@ZIF-8	1.71	0.00722	1.49	0.07	0.00031	1.60

Table S9. N_2 total adsorption quantities at 16 and 0.5 bar for ZIF-8 and the produced magIL@ZIF-8 in mol/kg, mol/cm^3 of available pore volume and normalized using ZIF-8 as reference. The data is reported for 303 K.

	16 bar			0.5 bar		
Sample	q_t (mol/kg)	q_t/V_p (mol/cm^3)	Normal. q_t/V_p	q_t (mol/kg)	q_t/V_p (mol/cm^3)	Normal. q_t/V_p
ZIF-8	1.59	0.00237	1.00	0.06	0.00009	1.00
$[C_4MIM]_2[NiCl_4]$@ZIF-8	0.74	0.00251	1.06	0.03	0.00010	1.07
$[C_4MIM]_2[MnCl_4]$@ZIF-8	0.53	0.00251	1.06	0.02	0.00010	1.04
$[C_4MIM]_2[CoCl_4]$@ZIF-8	0.84	0.00348	1.47	0.03	0.00014	1.54
$[C_4MIM]_2[Co(NCS)_4]$@ZIF-8	0.64	0.00232	0.98	0.02	0.00008	0.87
$[C_4MIM][FeCl_4]$@ZIF-8	0.77	0.00324	1.37	0.03	0.00014	1.47

S11. CO_2, CH_4 and N_2 Fitting Parameters for ZIF-8 and magIL@ZIF-8 Isotherms

Table S10. CO_2 fitting parameters from the experimental adsorption-desorption equilibrium isotherms at 303 K using Equation (5).

Samples	q_s	b	t
ZIF-8	8.090	0.083	3.372
$[C_4MIM]_2[NiCl_4]$@ZIF-8	5.168	0.082	2.308
$[C_4MIM]_2[MnCl_4]$@ZIF-8	3.780	0.089	1.529
$[C_4MIM]_2[CoCl_4]$@ZIF-8	5.345	0.087	2.734
$[C_4MIM]_2[Co(NCS)_4]$@ZIF-8	4.020	0.082	2.673
$[C_4MIM][FeCl_4]$@ZIF-8	4.763	0.088	2.273

Table S11. CH_4 fitting parameters from the experimental adsorption-desorption equilibrium isotherms at 303 K using Equation (5).

Samples	q_s	b	t
ZIF-8	6.876	0.038	1.508
$[C_4MIM]_2[NiCl_4]$@ZIF-8	3.783	0.041	1.399
$[C_4MIM]_2[MnCl_4]$@ZIF-8	1.924	0.052	1.632
$[C_4MIM]_2[CoCl_4]$@ZIF-8	3.902	0.045	1.425
$[C_4MIM]_2[Co(NCS)_4]$@ZIF-8	3.938	0.035	1.099
$[C_4MIM][FeCl_4]$@ZIF-8	3.648	0.041	1.319

Table S12. N_2 fitting parameters from the experimental adsorption-desorption equilibrium isotherms at 303 K using Equation (5).

Samples	q_s	b	t
ZIF-8	3.646	0.031	1.824
$[C_4MIM]_2[NiCl_4]$@ZIF-8	3.339	0.017	1.072
$[C_4MIM]_2[MnCl_4]$@ZIF-8	2.233	0.019	0.993
$[C_4MIM]_2[CoCl_4]$@ZIF-8	2.861	0.022	1.307
$[C_4MIM]_2[Co(NCS)_4]$@ZIF-8	2.358	0.020	1.255
$[C_4MIM][FeCl_4]$@ZIF-8	3.829	0.015	1.009
